# The activity of pyrazolo[4,3-*e*][1,2,4]triazine and pyrazolo[4,3-*e*]tetrazolo[1,5-*b*][1,2,4]triazine sulphonamide derivatives in monolayer and spheroid breast cancer cell cultures

**DOI:** 10.1080/14756366.2024.2343352

**Published:** 2024-05-03

**Authors:** Anna Szymanowska, Dominika Radomska, Robert Czarnomysy, Mariusz Mojzych, Katarzyna Kotwica-Mojzych, Krzysztof Bielawski, Anna Bielawska

**Affiliations:** a Department of Biotechnology, Medical University of Bialystok, Bialystok, Poland; b Department of Experimental Therapeutics, The University of Texas MD Anderson Cancer Center, Houston, TX, USA; c Department of Synthesis and Technology of Drugs, Medical University of Bialystok, Bialystok, Poland; d Department of Chemistry, Siedlce University of Natural Sciences and Humanities, Siedlce, Poland; e Laboratory of Experimental Cytology, Medical University of Lublin, Lublin, Poland

**Keywords:** Pyrazolo[43-*e*]tetrazolo[15-*b*][1,2,4]triazine, pyrazolo[43-*e*][1,2,4]triazine, anticancer, sulphonamide, spheroid

## Abstract

In the last decade, an increasing interest in compounds containing pyrazolo[4,3-*e*][1,2,4]triazine moiety is observed. Therefore, the aim of the research was to synthesise a novel sulphonyl pyrazolo[4,3-*e*][1,2,4]triazines (**2a**, **2b**) and pyrazolo[4,3-*e*]tetrazolo[1,5-*b*][1,2,4]triazine sulphonamide derivatives (**3a**, **3b**) to assess their anticancer activity. The MTT assay showed that **2a**, **2b**, **3a**, **3b** have stronger cytotoxic activity than cisplatin in both breast cancer cells (MCF-7 and MDA-MB-231) and exhibited weaker effect on normal breast cells (MCF-10A). The obtained results showed that the most active compound **3b** increased apoptosis via caspase 9, caspase 8, and caspase 3/7. It is worth to note that compound **3b** suppressed NF-κB expression and promoted p53, Bax, and ROS which play important role in activation of apoptosis. Moreover, our results confirmed that compound **3b** triggers autophagy through increased formation of autophagosomes, expression of beclin-1 and mTOR inhibition. Thus, our study defines a possible mechanism underlying **3b**-induced anti-cancer activity against breast cancer cell lines.

## Introduction

Over the past 20 years, the number of new breast cancer cases has increased from 1 002 000 to 2 261 419^1^^,^^2^. The rapid increase in the incidence of breast cancer causes it one of the most diagnosed cancer. Moreover, breast cancer mortality has raised from 459 000 deaths in 2000 to 684 996 in 2020^1^^,^^2^. The growth in both mortality and morbidity of this disease may be related with heterogeneous nature of breast cancer tumours, associated with diverse gene expression. Variation of neoplasms results in patient’s responsiveness to chemotherapy^3^.

According to the literature, MCF-7 and MDA-MB-231 are the most widely used breast cancer cell lines in *in vitro* studies of anticancer activity of novel compounds. MDA-MB-231 are grouped as triple negative B – basal b (ER(−), PR(−), and HER2(−)) and EGFR (+) cell lines, while MCF-7 is characterised as luminal a (ER(+), PR(+), and HER2(−))^3^^,^^4^. Moreover, invasive MDA-MB-231 cells use significantly more energy from aerobic glycolysis than non-invasive MCF-7. Altered metabolism of glucose by MDA-MB-231 is an adaptation to hypoxia. In both cell lines, glucose intake is decreased. Although MCF-7 cells have definitely lower glucose consumption under aerobic condition than MDA-MB-231^5^.

Chemotherapy still plays crucial role in cancer treatment. Hence, the research, development and synthesis of new, potential anticancer agents with appropriate effectiveness, selectivity, and specificity of action are of prime importance. The inventive process of designing new compounds with biological activity against cancer cells requires understanding the molecular mechanism of neoplasm development. In the recent years, intriguing role in the preclinical research plays heterocyclic scaffold. Wide interest in this chemical structure is associated with progress of the new synthetic routes that allow to receive active pharmaceutical compounds with high biological activity and low toxicity. Statistics shows that heterocycles with at least one nitrogen atom in the five and six membered ring have tremendous part in drug discovery^6^^,^^7^. Among them, triazine derivatives are worth to pay attention. Analysis of the published literature demonstrated potential antitumor activity of 1,2,4-triazine isomer. In recent years, 1,2,4-triazine derivatives and its benzo- and heterofused derivatives were designed and synthesised as potential anticancer drugs^8–16^. In addition to their anticancer activity, 1,2,4-triazines have unique biological properties including: antibacterial activity, antiviral, anti-inflammatory, antihypertensive, antidiabetic, etc. One of the derivatives, remdesivir has been approved by FDA in May 2020 in emergency treatment patients having severe symptoms of COVID-19^17^.

Important strategy in discovery of novel chemotherapeutics is combining pharmacophore group with anticancer compounds. This combination allows to increased biological activity by acting on multiple molecular targets, minimising drug resistance and side effects, and improving pharmacokinetic properties^18^^,^^19^. One of the most well-known pharmacophore group is sulphonamide group which is found in many medicine drugs. Their tremendous medical importance results from the pleiotropic effect documented in the literature which include anticancer activity ^20–23^. Therefore, it seems reasonable to combine sulphonamide group with triazine derivatives to increase biological activity of nitrogen containing compounds.

In continuation of our efforts in search of new effective anticancer agents, we synthesised new enantiomeric pyrazolo[4,3-*e*][1,2,4]triazine sulphonamides (**2a**,**b**) as pivotal intermediates for the preparation of novel pyrazolo[4,3-*e*]tetrazolo[1,5-*b*][1,2,4]triazine sulphonamide derivatives (**3a**,**b**) and evaluated their *in vitro* anticancer potential against MCF-7 and MDA-MB-231 cell lines. The promising activity obtained, prompted us to investigate their role in the apoptosis and autophagy.

## Materials and methods

### Synthesis

#### General

The melting points of the synthesised compounds were measured on Mel-Temp.^1^ H and ^13^C NMR spectra were measured in deuterated solvents on a Varian instrument (400 MHz for ^1^H and 100 MHz for ^13^C, Siedlce, Poland). The chemical shift values (*δ*) in both spectra are presented in ppm (part per million) and coupling constants (J) in Hz (Hertz). Tetramethylsilane (TMS) was used as internal standard. The measure of signal intensity in ^1^H NMR spectra is proportional to the number of protons which caused the signal. Molecular weights of compounds (**2a**, **2b**, **3a**, **3b**) were evaluated by electrospray ionisation mass spectrometry (ESI/MS) on an Agilent Technologies 6538 UHD Accurate Mass Q-TOF LC/MS (Agilent Technologies, Inc., Santa Clara, CA). Elemental compositions are within ±0.4% of the calculated values. Compound **1** was synthesised according to the methods described in the literature^24^.

#### Synthesis of sulphonyl derivatives 2a,b

Compound **1** (200 mg, 0.515 mmol) was dissolved in anhydrous acetonitrile (10 mL) (Sigma Chemical Co., St. Louis, MO) and the corresponding amine (1.003 mmol) (Sigma Chemical Co., St. Louis, MO) was added. The obtained solution was stirred at room temperature until the substrate disappeared (control TLC) plates. When the reaction was completed, the solvent was evaporated and the remaining precipitate was purified by chromatography column using a mixture of methylene chloride:ethanol (50:1) as an eluent (Sigma Chemical Co., St. Louis, MO).

(S)-*N*-(1-Hydroxy-3-phenylpropan-2-yl)-4-(3-methyl-5-(methylsulfonyl)-1*H*-pyrazolo[4,3-*e*][1,2,4]triazin-1-yl)benzenesulfonamide (**2a**): yield 90%. Melting point: 130–134 °C. ^1^H NMR (DMSO): 2.38–2.43 (m, 1H), 2.75 (s, 3H), 2.79–2.83 (m, 1H), 3.20–3.28 (m, 1H), 3.30–3.38 (m, 2H), 3.56 (s, 3H), 4.85 (t, 1H, *J* = 6.4 Hz, OH exchanged with D_2_O), 6.95–7.08 (m, 5H), 7.78 (d, 2H, *J* = 8.8 Hz), 8.28 (d, 2H, *J* = 8.8 Hz). ^13^C NMR (DMSO): 11.12, 37.08, 40.82, 57.33, 63.27, 119.83, 126.03, 127.97, 128.08, 129.15, 138.15, 138.31, 139.69, 139.90, 145.98, 148.23, 160.95; HRMS (ESI, *m/z*) Calcd. for C_21_H_22_N_6_O_4_S_2_ [M^+^+H] 503.11659. Found [M^+^+H] 503.11719. Anal. Calcd. for C_21_H_22_N_6_O_4_S_2_: C, 50.19; H, 4.41; N, 16.72. Found: C, 50.10; H, 4.46; N, 16.50.

(*R*)-*N*-(1-Hydroxy-3-phenylpropan-2-yl)-4-(3-methyl-5-(methylsulfonyl)-1*H*-pyrazolo[4,3-*e*][1,2,4]triazin-1-yl)benzenesulfonamide (**2b**): yield 92%. Melting point: 124–128 °C. ^1^H NMR (DMSO): 2.39–2.44 (m, 1H), 2.76 (s, 3H), 2.79–2.84 (m, 1H), 3.20–3.28 (m, 1H), 3.30–3.38 (m, 2H), 3.63 (s, 3H), 4.85 (t, 1H, *J* = 6.4 Hz, OH exchanged with D_2_O), 6.96–7.10 (m, 5H), 7.79 (d, 2H, *J* = 8.8 Hz), 8.29 (d, 2H, *J* = 8.8 Hz).^13^ C NMR (DMSO): 11.12, 37.08, 40.81, 57.33, 63.26, 119.83, 126.03, 127.97, 128.07, 129.15, 138.15, 138.31, 139.69, 139.90, 145.97, 148.23, 160.94; HRMS (ESI, *m/z*) Calcd. for C_21_H_22_N_6_O_4_S_2_ [M^+^+H] 503.11659. Found [M^+^+H] 503.11730. Anal. Calcd. for C_21_H_22_N_6_O_4_S_2_: C, 50.19; H, 4.41; N, 16.72. Found: C, 50.00; H, 4.53; N, 16.52.

#### Synthesis of pyrazolo[4,3-e]tetrazolo[1,5-b][1,2,4]triazine sulphonamide 3a,b

The appropriate compound **2a** or **2b** (0.29 mmol) was dissolved in anhydrous ethanol (25 mL), sodium azide (22 mg, 0.35 mmol) (both of Sigma Chemical Co., St. Louis, MO) was added to the solution. The reaction mixture was refluxed. The progress of the reaction was controlled by TLC until all substrate reacted. Then solvent was evaporated on evaporator and the final compound (**3a** or **3b**) was purified on column chromatography using methylene chloride:ethanol (100:1) (Sigma Chemical Co., St. Louis, MO).

(*S*)-*N*-(1-Hydroxy-3-phenylpropan-2-yl)-4-(7-methyl-5*H*-pyrazolo[4,3-*e*]tetrazolo[1,5-*b*][1,2,4]triazin-6-yl)benzenesulfonamide (**3a**): yield 82%. m.p. 172–176 °C.

^1^H NMR (MeOH-d_4_): 2.50–2.55 (m, 1H), 2.86 (s, 3H), 2.90–2.95 (m, 1H), 3.46–3.50 (m, 2H), 3.55–3.62 (m, 1H), 4.28 (t, 1H, *J* = 6.4 Hz, OH exchanged with D_2_O), 7.02–7.12 (m, 5H), 7.82 (d, 2H, *J* = 8.8 Hz), 8.24 (d, 2H, *J* = 8.8 Hz). ^13^C NMR (DMSO): 11.10, 37.09, 57.34, 63.33, 118.75, 126.08, 128.12, 128.17, 129.17, 138.34, 139.07, 139.50, 142.03, 146.98, 147.20, 147.77; HRMS (ESI, *m/z*) Calcd. for C_20_H_20_N_9_O_3_S [M^+^+H] 466.14043. Found [M^+^+H] 466.14079. Anal. Calcd. for C_20_H_19_N_9_O_3_S: C, 51.61; H, 4.11; N, 27.08. Found: C, 51.78; H, 4.39; N, 26.80.

(*R*)-*N*-(1-hydroxy-3-phenylpropan-2-yl)-4-(7-methyl-5*H*-pyrazolo[4,3-*e*]tetrazolo[1,5-*b*][1,2,4]triazin-6-yl)benzenesulfonamide (**3b**): yield 86%. m.p. 170–172 °C.

^1^H NMR (CDCl_3_): 2.73–2.76 (m, 1H), 2.83–2.85 (m, 1H), 3.52–3.55 (m, 2H), 3.57–3.61 (m, 1H), 3.68–3.70 (m, 1H), 4.80 (t, 1H, *J* = 6.4 Hz, OH exchanged with D_2_O), 7.15–7.19 (m, 5H), 7.88 (d, 2H, *J* = 8.8 Hz), 8.32 (d, 2H, *J* = 8.8 Hz). ^13^C NMR (DMSO): 11.09, 37.08, 57.34, 63.32, 118.75, 126.07, 128.11, 128.16, 129.17, 138.34, 139.06, 139.49, 142.03, 146.97, 147.19, 147.76; HRMS (ESI, *m/z*) Calcd. for C_20_H_20_N_9_O_3_S [M^+^+H] 466.14043. Found [M^+^+H] 466.14055. Anal. Calcd. for C_20_H_19_N_9_O_3_S: C, 51.61; H, 4.11; N, 27.08. Found: C, 51.70; H, 4.35; N, 26.84.

### Cell culture of breast cancer cells (MCF-7, MDA-MB-231), normal breast cells (MCF-10a)

MCF-7, MDA-MB-231 human breast cancer cells were purchased from ATCC (Manassas, VA) and cultured DMEM (Corning, Kennebunk, ME) with 10% heat-inactivated foetal bovine serum (Eurx, Gdańsk, Poland) and 100 μg/mL antibiotics (penicillin/streptomycin) (Corning, Kennebunk, ME). Both cell lines were incubated under optimal condition, i.e. 37 °C in a humidified atmosphere (90–95%) containing 5% of CO_2_. To perform experiments, cells were plated in six-well plates until they reach 80% of confluency.

MCF-10A normal human breast cancer cells were provided by ATCC and cultured in MEGM supplemented with Mammary Epithelial Cell Growth Kit (H-insulin, l-glutamine, epinephrine, apo-transferrin, rH-TGFα, extract P, and hydrocortisone hemisuccinate) and 10% of FBS and 100 µg/mL antibiotics (penicillin and streptomycin). MCF-10A was incubated under the same conditions as cancer cells.

### Viability assay

The MTT viability assay was done as described previously^25^^,^^26^. In brief, cells were incubated with novel synthesised compounds **2a**, **2b**, **3a**, **3c** and reference drug cisplatin. Following 24-h incubation with tested compounds, the medium was discarded from the cell cultures. Then, into each well with cells was added 1 mL of PBS and 50 µL of MTT solution (5 mg per mL). Cells were incubated at 37 °C and 5% CO_2_ in humidified atmosphere for a defined time appropriate for each cell line. After incubation, the solution above the cells was removed and obtained purple formazan crystals were dissolved in 1 mL DMSO. The absorbance was read at OD = 570 nm on Evolution 201 Spectrophotometer (Thermo Scientific, Waltham, MA).

### Apoptosis

MCF-7 and MDA-MB-231 cell lines were incubated with or without novel compounds (**2b**, **3b**) and cisplatin at concentration 0.25 µM and 0.5 µM for 24 h and stained with Annexin V-FITC/PI. The analysis was performed using flow cytometry Apoptosis Detection Kit II (BD Biosciences, San Diego, CA) as described previously^27^.

### Total ROS activity

The effect of **3b** and cisplatin on ROS induced cellular damage of two breast cancer cells (MCF-7 and MDA-MB-231) was performer as described previously^27^. In brief, cells were washed two times with cold PBS solution and incubated in a solution of ROS Green dye in Assay Buffer and incubated for 1 h in optimal cell growth parameters, protected from light. After incubation, cells were washed with assay buffer. Subsequently, fluorescence emission intensities at 490 nm excitation and 520 nm emission were recorded in 500 μL assay buffer using flow cytometer BD FACSCanto II flow cytometer and FACSDiva software (BD Biosciences Systems, San Jose, CA).

### Activity of caspase-9, -10, and -3/7

MCF-7 and MDA-MB-231 cells were plated in six-well plates at density 1 × 10^6^ and cultured in DMEM for 24 h. Following incubation, cells were treated with compound **3b** and cisplatin at concentration 0.25 µM and 0.5 µM. Then to identify the involvement of caspase family in inducing apoptosis, cells were pre-treated with Z-VAD-FMK (100 µM) 2 h before adding compounds^28^. The activation of caspases 9, 10, and 3/7 was detected as described before^14^.

### Autophagy

The effect of novel compound **3b** at 0.25 µM and 0.5 µM concentration on the induction of autophagy in MCF-7 and MDA-MB-231 pre-treated 1 h with or not autophagy inhibitor 3-MA for 1 h (3-methyladenine, Sigma-Aldrich, St. Louis, MO) at 1 mM concentration^29^ was assessed by ICT’s Autophagy Assay, Red using a flow cytometer. The test was performed as previously^25^. Briefly, cells were washed with PBS and suspended in 490 µL of PBS. To each probe, 10 µL of diluted Autophagy Probe, Red solution was added. Prepared samples were incubated at 37 °C for 60 min. Following incubation, cells were suspended in diluted cellular assay buffer and centrifuged. Then, supernatant was removed, and cells were resuspended in diluted cellular assay buffer and centrifuged again. This step was repeated three times. Finally, cell pellet was resuspended in 0.5 mL of diluted cellular assay buffer. The analysis was performed with a flow cytometer (excitation/emission 590 nm/620 nm).

### p53, NF-κB, mTOR, and Bax

To evaluate the molecular mechanism of induction of apoptosis of tested compounds, the level of p53, NF-κB, mTOR, Bax, and beclin-1 was measured using appropriate antibody according to the manufacturer’s instruction. In brief, tryspinised cells were centrifuged, then supernatant was discarded, and cells were suspended in 4% FA (formaldehyde). Prepared samples were incubated 15 min at RT. Following incubation, the 2.5 mL PBS was added to each sample and centrifuged. Then, supernatant was discarded, and cells were resuspended in 90% ice-cold methanol to perform permeabilisation and left for 1 h in ice. To remove methanol, cells were rinsed with excess PBS by centrifugation. Following centrifugation, the supernatant was discarded. Then, cells were resuspended in 100 µL of appropriate diluted (1:100 in PBS) antibody conjugate (p53 (7F5) Rabbit mAb – Alexa Fluor 488 Conjugate #5429 Cell Signalling Technology, Danvers, MA; NF-κB p65 (D14E12) XP Rabbit mAb – Alexa Fluor 647 Conjugate #8801 Cell Signalling Technology, Danvers, MA; mTOR (7C10) Rabbit mAB – Alexa Fluor 647 #5048 Cell Signalling Technology, Danvers, MA; FITC Anti-Bax antibody (T22-A) #ab139543, Abcam, Cambridge, UK) and incubated 1 h in the dark at RT. Afterwards, cells were washed by centrifugation in 2 mL of incubation buffer. Finally, supernatant was removed and 300 µL PBS was added to each sample and pipetted. The analysis was performed by flow cytometry (BD FACSCanto II, Becton Dickinson Biosciences Systems, San Jose, CA) using FACSDiva software ver. 6.1.3.

### Beclin-1

The fixation and permeabilisation were performed as described above. Cells were resuspended in 100 µL of diluted primary antibody (1:100 in PBS, anti-beclin 1 antibody (2A4) #ab114071, Abcam, Cambridge, UK) and incubated 30 min in the dark at RT. The cells were washed three times by centrifugation in PBS. Then to each probe, 100 µL of diluted fluorochrome-labelled secondary antibody (1:100 in PBS; goat anti-mouse IgG H&L – Alexa Fluor 488 #ab150113, Abcam, Cambridge, UK) was added and incubated 30 min in dark at RT. Then, cells were rinsed with 2.5 mL PBS. In the last step, supernatant was discarded and cells were resuspended in 300 µL PBS and pipetted. The analysis was carried out by flow cytometry.

### 3D spheroids of both cancer cell lines: MCF-7 and MDA-MB-231

The 3D cell culture was carried out with 6-Well Bio-Assembler Kit in accordance with the manufacturer’s instructions (Greiner Bio-One GmbH, Frickenhausen, Germany). First, MCF-7 and MDA-MB-231 were cultured in 2D using procedure and supplied to 80% confluency and treated with nanoshuttle at concentration of 1 µL/10 000 cell and incubated overnight. Following incubation, the medium was removed, and the cells were detached by trypsin–EDTA solution and resuspended in DMEM. The cells were counted using Scepter 2.0 Handheld Automated Cell Counter (Merck, Darmstadt, Germany) and plated at the concentration 1 × 10^6^ cells per well in six-well plate. Then, cells were gently tilted in the flask to evenly distribute the nanoparticle and incubated (37 °C, 5% CO_2_, 95–98% humidity) for 1 h with magnet on the top of the plate. The cells self-assemble to spheroids. The obtained 3D cell culture was incubated until analysis^30^.

The spheroids were incubated with tested drugs (**2b**, **3b**) and cisplatin at 0.25 µM and 0.5 µM concentrations. The visualisation of the morphological changes in 3D cell culture of MCF-7 and MDA-MB-231 was evaluated using microscope (Nikon Eclipse Ti, Tokyo, Japan).

### Statistical analysis

The mean ± standard deviation (SD) for all data is presented. One-way and two-way analysis of variance (ANOVA) was conducted followed by Dunnett’s test. GraphPad 8.0.1 Prism software (GraphPad Software, San Diego, CA) was used for calculation. The analysis of spheroids area was carried out using ImageJ software from the NIH (Bethesda, MD).

## Results

### Chemistry

**Scheme 1.**
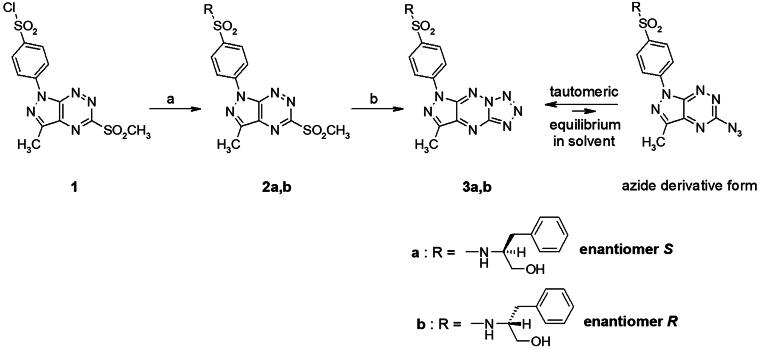
Reagents and conditions: (a) appropriate phenylalaninol derivative, acetonitrile, rt; (b) NaN_3_, EtOH, reflux.

Sulphonamides **2a,b** and **3a,b** were prepared using previously published procedure^12–14^ and the synthetic route is briefly presented in Scheme 1. The synthesis was started with the preparation of the pivotal intermediate benzenesulfonyl chloride (**1**), which was obtained by the chlorosulfonation reaction using our previously published procedure^12–14^^,^^24^. The preparation of the sulphonamide intermediates (**2a,b**) was performed via *N*-sulfonation reactions of compound **1** with appropriate enantiomeric phenylalaninol derivative in acetonitrile at room temperature (Scheme 1). Further, these compounds **2a,b** were reacted with sodium azide in ethanol to afford the final tricyclic sulphonamides **3a,b**. The structures of compounds **2a,b/3a,b** were confirmed by spectroscopic techniques. In the ^1^H NMR spectrum, the products **2a,b** showed similar chemical shift values for aromatic protons to those of compound **1** but slightly shifted upfield. Additionally, at 7.00–7.10 ppm, a multiplet was observed corresponding to the five protons of the phenyl ring present in the amine moiety. Analogous to our previous research results^12^^,^^14^, tautomeric equilibrium between azide derivatives and appropriate fused tetrazoles was observed in the ^1^H NMR spectra. We have observed additional low-intensity signals on ^1^H NMR spectra that correspond to the suitable azido derivatives forming in solution from tetrazole derivatives.

### Biological studies

**Figure 1.**
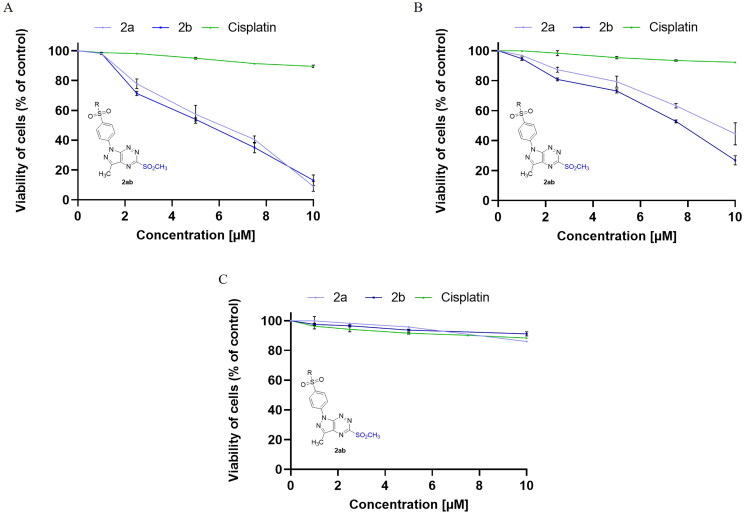
Viability of MCF-7 (A), MDA-MB-231 (B) breast cancer cells, and MCF-10A (C) normal breast cells incubated for 24 h with different concentrations of **2a**, **2b** and cisplatin. Mean values ± SD from three independent experiment (*n* = 3) done in duplicate are presented.

**Figure 2.**
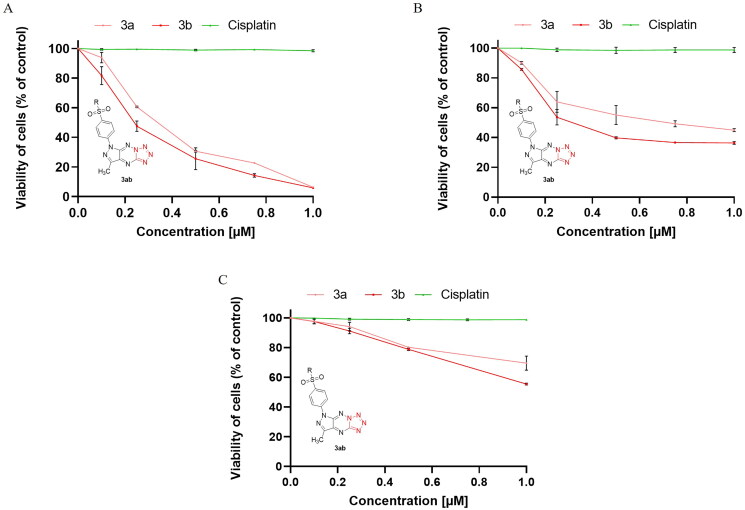
Viability of MCF-7 (A), MDA-MB-231 (B) breast cancer cells, and MCF-10A (C) normal breast cells incubated for 24 h with different concentrations of **3a**, **3b** and cisplatin. Mean values ± SD from three independent experiment (*n* = 3) done in duplicate are presented.

In a continuation of our previous studies on bioactive compounds of pyrazolo[4,3-*e*]tetrazolo[1,5-*b*][1,2,4]triazine derivatives^12^^,^^14^, the cytotoxic activity of enantiomeric sulphones (**2a**,**2b**) and their appropriate tricyclic derivatives (**3a**,**3b**) of (*S*)- and (*R*)-phenylalaninol was investigated against two breast cancer cell lines (MCF-7 and MDA-MB-231) and normal breast cell line (MCF-10A). In Figures 1 and 2, it is shown that survival of MCF-7 and MDA-MB-231 was significantly inhibited after 24 h incubation with **2a**, **2b**, **3a** and **3b** in concentration-dependent manner. The results revealed the inhibitory concentrations (IC_50_) of **2a**, **2b**, **3a**, **3b** were 6.0 ± 0.38 µM, 5.5 ± 0.39 µM, 0.35 ± 0.08 µM, and 0.25 ± 0.07 µM (Table 1) in MCF-7 and 9.2 ± 0.78 µM, 7.8 ± 0.53 µM, 0.71 ± 0.14 µM, and 0.31 ± 0.14 µM in MDA-MB-231, respectively.

**Table 1.** The cytotoxic activity of new derivatives of pyrazolo[4,3-e][1,2,4]triazine.

Compound	Cytotoxicity IC_50_		
	MCF-7	MDA-MB-231	MCF-10A
**2a**	6.0 ± 0.38 µM	9.2 ± 0.78 µM	>10.0 µM
**2b**	5.5 ± 0.39 µM	7.8 ± 0.53 µM	>10.0 µM
**3a**	0.35 ± 0.08 µM	0.71 ± 0.14 µM	3.5 ± 0.18 µM
**3b**	0.25 ± 0.07 µM	0.31 ± 0.14 µM	2.3 ± 0.04 µM
**Cisplatin**	>10.0 µM	>10.0 µM	>10.0 µM

According to the outcomes, the cytotoxic effect of (*R*)-enantiomers (**2b**,**3b**) was slightly higher than (*S*)-enantiomers (**2a**,**3a**) in both breast cancer cell lines (MCF-7 and MDA-MB-231). The pyrazolo[4,3-*e*]tetrazolo[1,5-*b*][1,2,4]triazine derivatives (**3a, 3b**) have stronger cytotoxic activity against both cancer cell lines compared to the sulphonyl from which they were derived (**2a**,**b**). The **3b** inhibited MCF-7 and MDA-MB-231 cancer cell viability to the highest rate. Moreover, **3b** exerted weaker cytotoxic effect on normal breast cells (IC_50_ = 2.3 ± 0.04 µM) than on cancer cells. According to the previous studies, the IC_50_ value for cisplatin (used as reference drug) was 82 µM in MDA-MB-231 cell line ^31^, 93 µM in MCF-7 ^31^ and above 100 µM in MCF-10A ^25^ after 24 h incubation.

For further studies as the most potent compound, **3b** and its respective sulphonamide **2b** were selected. Their effect on the molecular mechanism of apoptosis and autophagy was evaluated.

**Figure 3.**
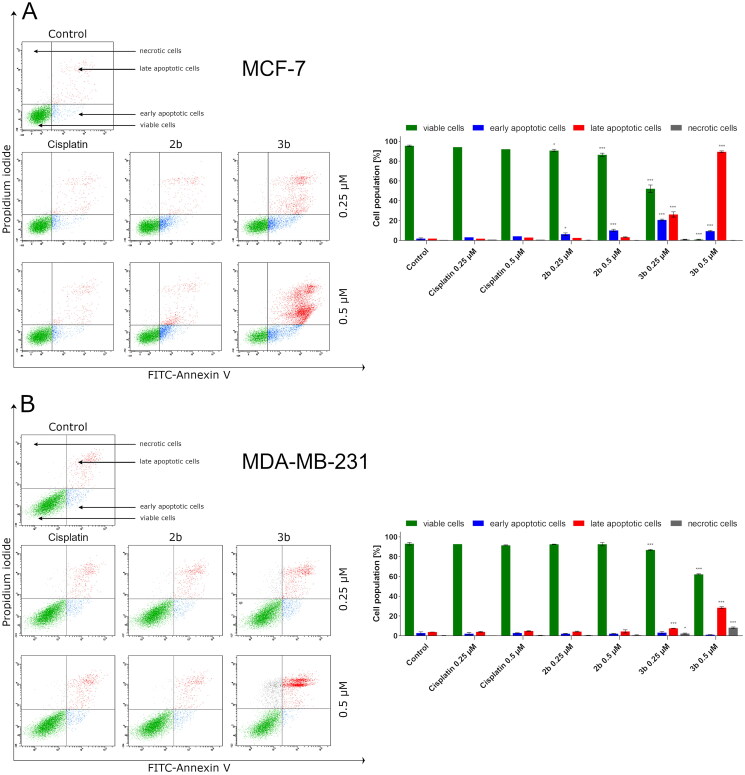
Flow cytometric analysis of induction of apoptosis in MCF-7 (A) and MDA-MB-231 (B) breast cancer cells incubated for 24 h with **2b**, **3b** and cisplatin (0.25 μM and 0.5 μM) stained with annexin V-FITC and propidium iodide. Mean percentage values from three independent experiments done in duplicate are presented. *p < 0.05 vs. control group and ***p < 0.001 vs. control group.

For quantitative determination of the apoptotic effects of **2b** and **3b** on MCF-7 and MDA-MB-231, flow cytometric assay was performed using double staining annexin V-FITC and propidium iodide (AV/PI). This study makes it possible to distinguish between four cell populations: viable cells, cells in early apoptosis, cells in late apoptosis, and necrotic cells. The results indicated that **3b** induced apoptosis in both breast cancer cell lines was much greater than its respective sulphonyl **2b**. It is intriguing that the number of apoptotic cells was related to the type of cell line. Compound **3b** at 0.25 µM concentration caused an increase in apoptotic cells to 50.6 ± 3.5% in MCF-7 cell line and 11.8 ± 1.3% in MDA-MB-231. Whereas at concentration of 0.5 µM, 98.9 ± 1.6% of early and late apoptotic cells were noted in MCF-7 and 29.5 ± 0.9% in MDA-MB-231. The poorer proapoptotic activity was observed in both breast cancer cell lines exposed to compound **2b**. This derivative of pyrazolo[4,3-*e*][1,2,4]triazine caused an elevation in the number of apoptotic cells to 9.8 ± 1.2% at 0.25 µM concentration in MCF-7 and 5.7 ± 0.6% in MDA-MB-231. At 0.5 µM concentration, 14.6 ± 1.7% of apoptotic cells were observed in MCF-7 and 5.8 ± 1.8% in MDA-MB-231. For the reference drug, 7.1 ± 0.9% apoptotic cells were noted after 24 h exposure at 0.5 µM in MCF-7 and 7.6 ± 0.6% in MDA-MB-231. As can be seen from Figure 3, tetrazole derivative (**3b**) had marked stronger proapoptotic effect against both breast cancer cell lines than its sulphonyl derivative (**2b**). Thus, all further experiments were only performed with the **3b**.

**Figure 4.**
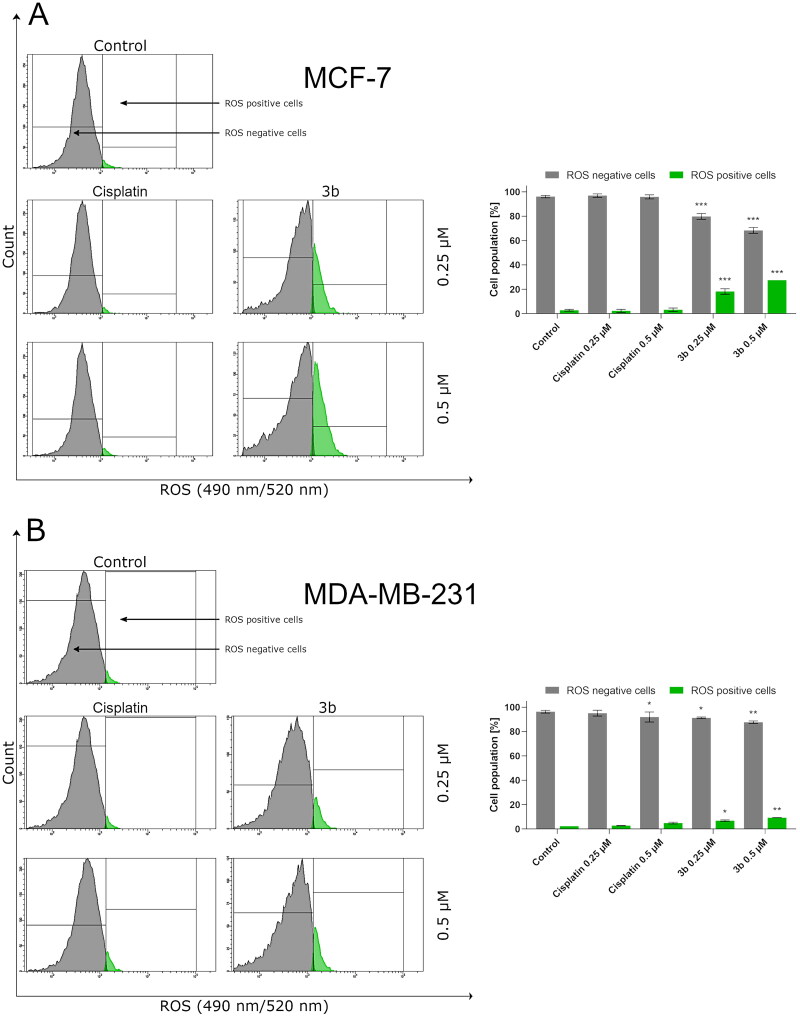
Flow cytometric analysis of ROS induction in MCF-7 (A) and MDA-MB-231 (B) breast cancer cells incubated for 24 h with **3b** and cisplatin (0.25 μM and 0.5 μM). Mean percentage values from three independent experiments done in duplicate are presented. *p < 0.05 vs. control group, **p < 0.01 vs. control group, ***p < 0.001 vs. control group.

Recent scientific reports show that one of the clue factors involved in the induction of cancerous transformation are reactive oxygen species – ROS^32^. These elements are essential for tumour cell proliferation survival, invasion, and metastasis. On the other hand, free radicals and compounds which lead to oxidative stress may induce PCD by triggering extrinsic or intrinsic pathway of apoptosis. Based on the dual role that ROS has, we evaluated the effect of **3b** on the oxidative stress in MCF-7 and MDA-MB-231 cells. Following incubation with **3b**, the flow cytometry revealed that **3b** increased ROS generation in a dose-dependent manner. In MCF-7 at 0.25 µM concentration, 19.8 ± 2.2% cells have generated ROS and at 0.5 µM 27.5 ± 1.3%. On MDA-MB-231, there was observed 7.0 ± 0.6% cells with ROS at 0.25 µM concentration and 9.4 ± 0.7% at 0.5 µM. It is worth mentioning that the number of cells with ROS was almost threefold higher in MCF-7 cell line than in MDA-MB-231 in both concentrations of tested compound (**3b**). The obtained results are shown in Figure 4.

**Figure 5.**
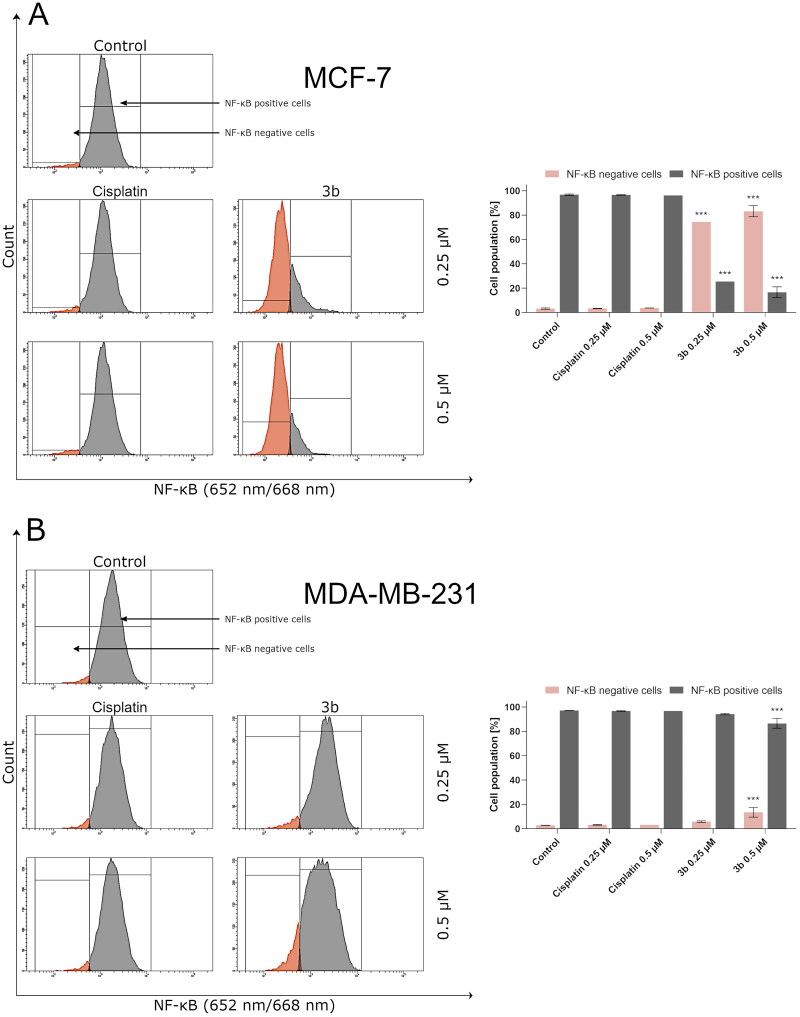
Anti-NF-κB antibody flow cytometric analysis of MCF-7 (A) and MDA-MB-231 (B) breast cancer cells compared to a negative control cell after 24 h of incubation with **3b** and cisplatin (0.25 μM and 0.5 μM). Mean percentage values from three independent experiments done in duplicate are presented. ***p < 0.001 vs. control group.

Moreover, the last data suggest that ROS can activate or deactivate nuclear factor κ light chain enhancer of activated B cells ^33^. In cancer cells, NF-κB is overactivated. This protein possesses antiapoptotic properties by stimulating expression of antiapoptotic proteins of Bcl-2 family and inhibiting the JNK signalling pathway^34–36^. In 2018, Huang and Xin demonstrated that intracellular ROS may inhibit signalling pathway associated with NF-κB^37^. During the flow cytometry analysis of activity of NF-κB in breast cancer cell lines incubated with tested compound, it was shown that pyrazolo[4,3-*e*]tetrazolo[1,5-*b*][1,2,4]triazine derivative (**3b)** leads to decreased number of cells with active NF-κB (Figure 5). In MCF-7 cells incubated 24 h with **3b** at concentration 0.25 µM, there was observed 25.5 ± 4.5% of cells with active NF-κB and at 0.5 µM 19.8 ± 4.4% while in control was 97.2 ± 0.6%. After 24 h exposure of MDA-MB-231 to **3b** at 0.25 µM and 0.5 µM, the percentage of cells with active NF-κB was 94.8 ± 0.6% and 89.6 ± 4.1%, respectively. Studies proved that **3b** decreased the activity of NF-κB in both cell lines. The effect was more noticeable in MCF-7 cell line.

**Figure 6.**
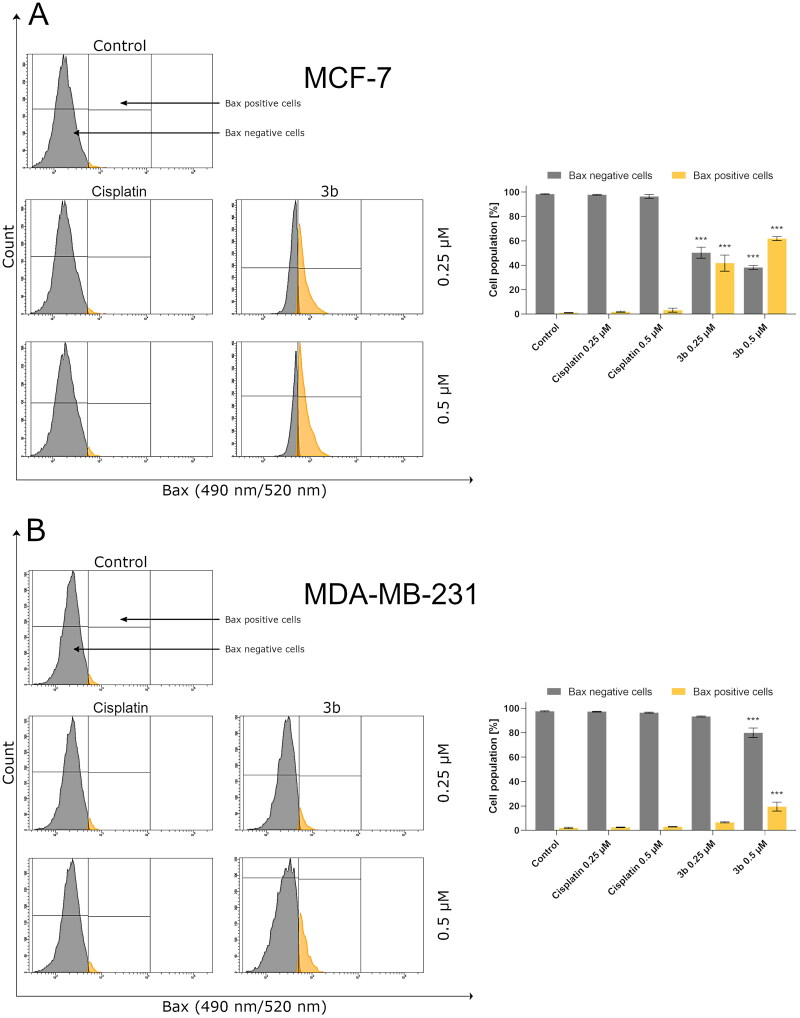
Anti-Bax antibody flow cytometric analysis of MCF-7 (A) and MDA-MB-231 (B) breast cancer cells compared to a negative control cell after 24 h of incubation with **3b** and cisplatin (0.25 μM and 0.5 μM). Mean percentage values from three independent experiments done in duplicate are presented. ***p < 0.001 vs. control group.

The intrinsic pathway of apoptosis could also be activated by ROS through an increase of protein Bax ^38^. This protein promotes apoptosis by creating pores in outer mitochondrial membrane and consequently increasing its permeability^39^. Thus, in the next step of the research, the effect of novel compound **3b** on Bax level in MCF-7 and MDA-MB-231 cells was evaluated. The number of cells with active Bax after exposition to cisplatin (24 h) was at the control level in both breast cancer cells. In contrast, incubation with **3b** resulted in dose-dependent increase of Bax either in MCF-7 and MDA-MB-231 as shown in Figure 6. The incubation with **3b** (0.25 µM) increased number of cells with active Bax to 46.4 ± 6.6% in MCF-7 and 6.3 ± 0.4% in MDA-MB-231. After exposure of MCF-7 and MDA-MB-231 to higher concentrations, this value increased to 63.0 ± 1.6% and 16.9 ± 3.7%, respectively.

**Figure 7.**
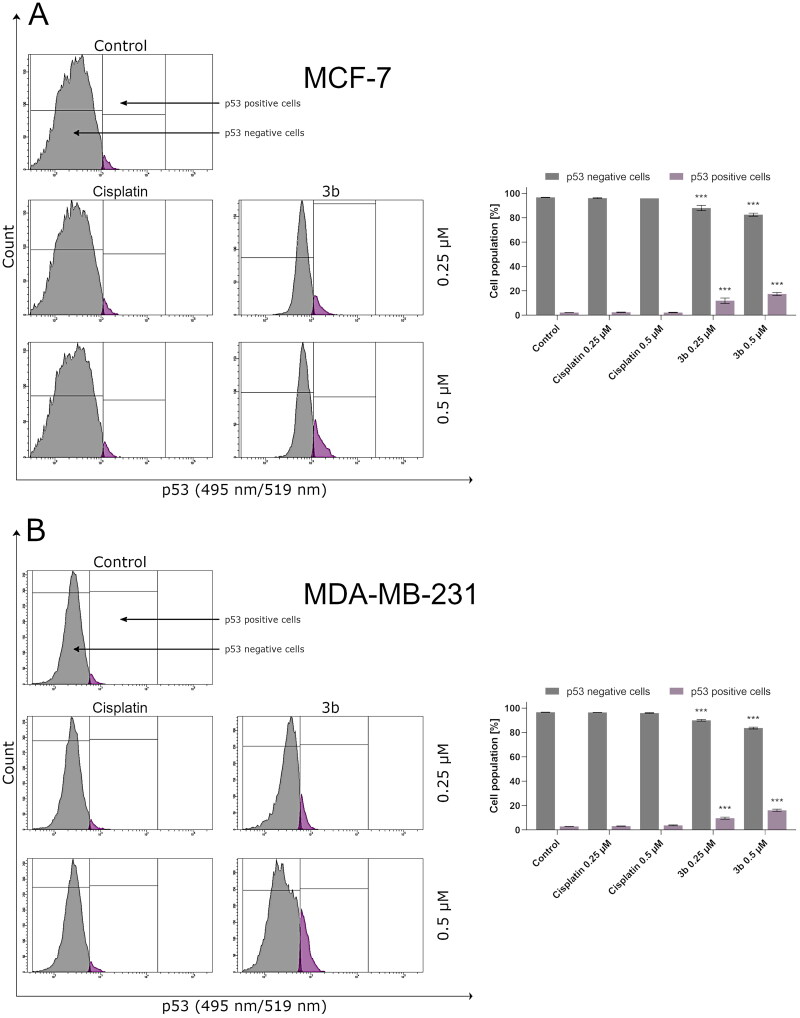
Anti-p53 antibody flow cytometric analysis of MCF-7 (A) and MDA-MB-231 (B) breast cancer cells compared to a negative control cell after 24 h of incubation with **3b** and cisplatin (0.25 μM and 0.5 μM). Mean percentage values from three independent experiments done in duplicate are presented. ***p < 0.001 vs. control group.

In addition, tumour suppressor p53 is a regulator of Bax gene expression *in vitro* and *in vivo*^40^. According to the literature, the p53 protein activates the transcription genes involved in cell cycle regulation, apoptosis, and DNA repair in response to various stress factors. This mechanism ensures stability of genetic materials and prevents division of mutated cells and in consequence development of cancer^41^. In human, invasive breast cancer *TP53* is most common mutated gene^42^. This mutation is associated with metastasis and poor overall patient’s survival. It is worth noting that in 80% of triple negative breast cancer cases, there is mutation in *TP53*. Therefore, activation of p53 may be an interesting molecular target of anticancer therapy. In this study, we have proved that novel compound **3b** activates p53 in MCF-7 and MDA-MB-231 cell lines (Figure 7).

**Figure 8.**
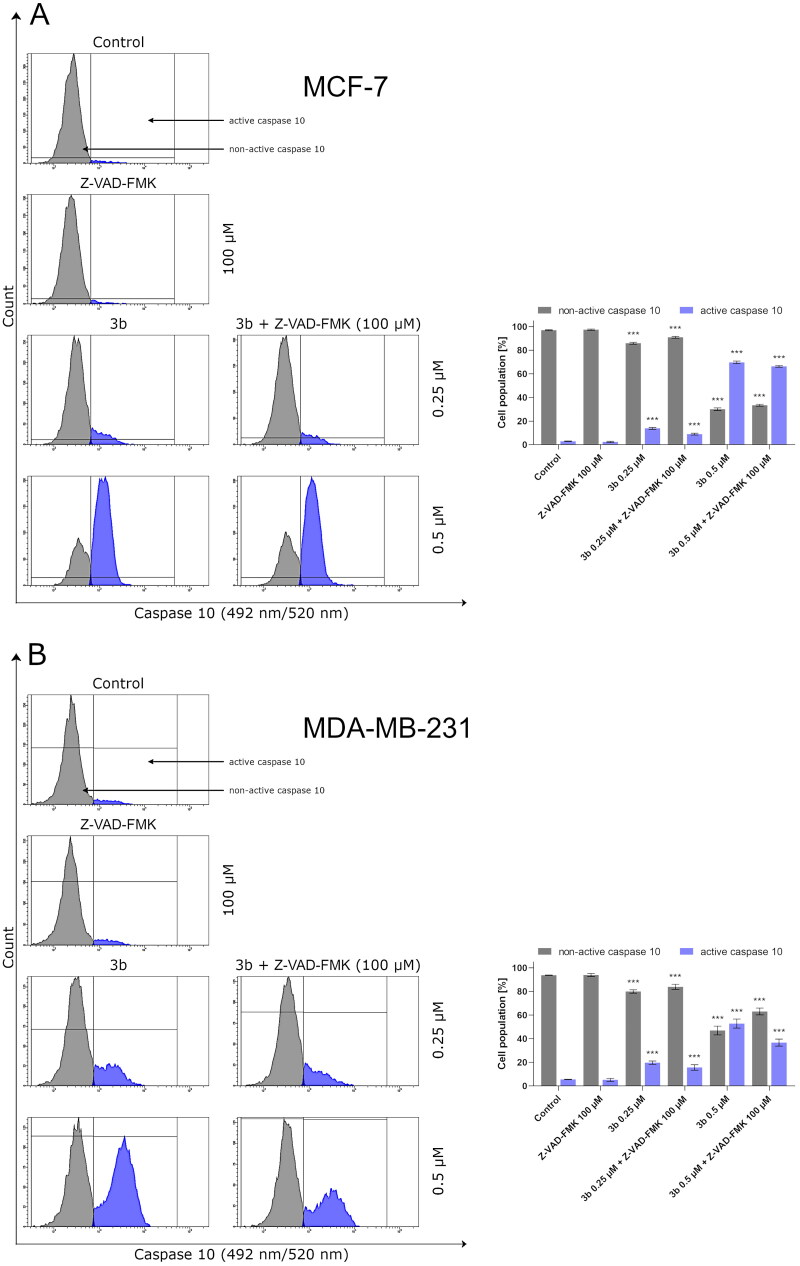
The activity of caspase 10 in MCF-7 (A) and MDA-MB-231 (B) breast cancer cells incubated with **3b** (0.25 μM and 0.5 μM) in the absence and presence of Z-VAD-FMK (100 μM) for 24 h. Mean percentage values from three independent experiments done in duplicate are presented. ***p < 0.001 vs. control group.

**Figure 9.**
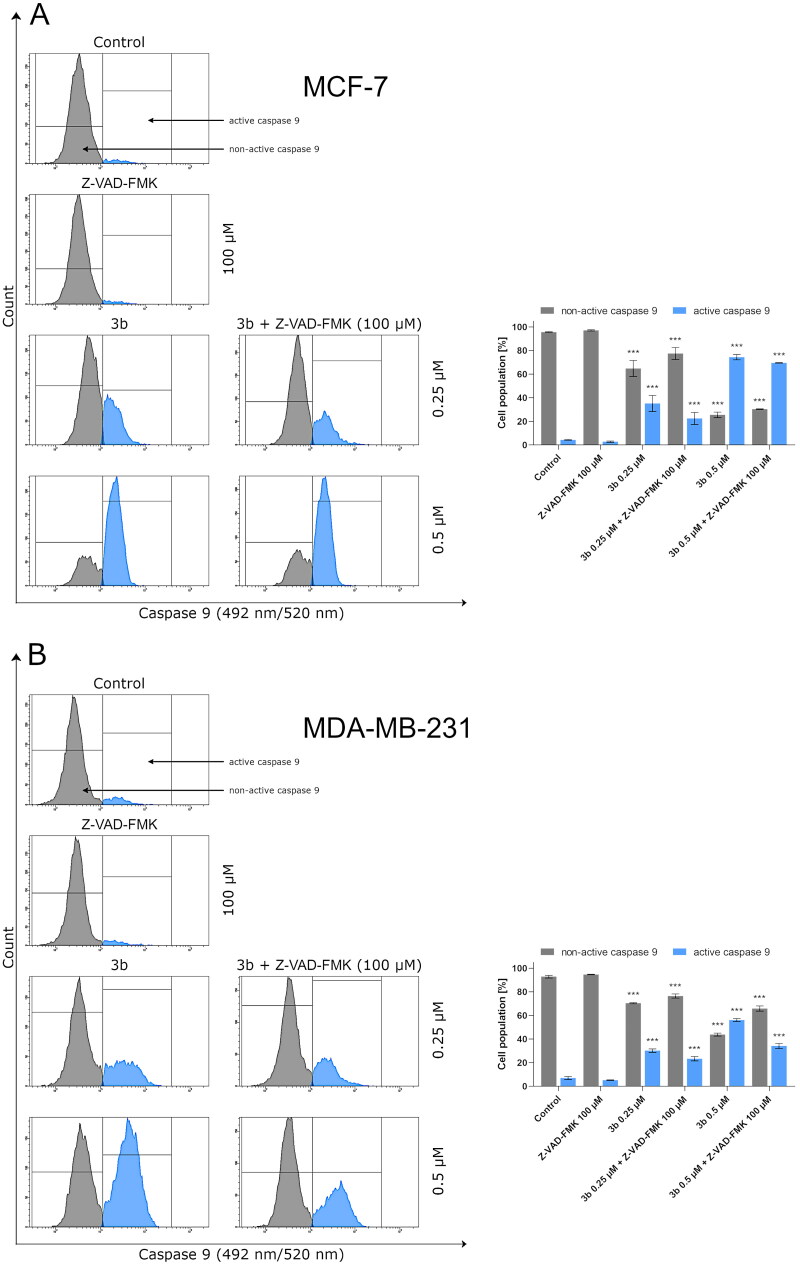
The activity of caspase 9 in MCF-7 (A) and MDA-MB-231 (B) breast cancer cells incubated with **3b** (0.25 μM and 0.5 μM) in the absence and presence of Z-VAD-FMK (100 μM) for 24 h. Mean percentage values from three independent experiments done in duplicate are presented. ***p < 0.001 vs. control group.

**Figure 10.**
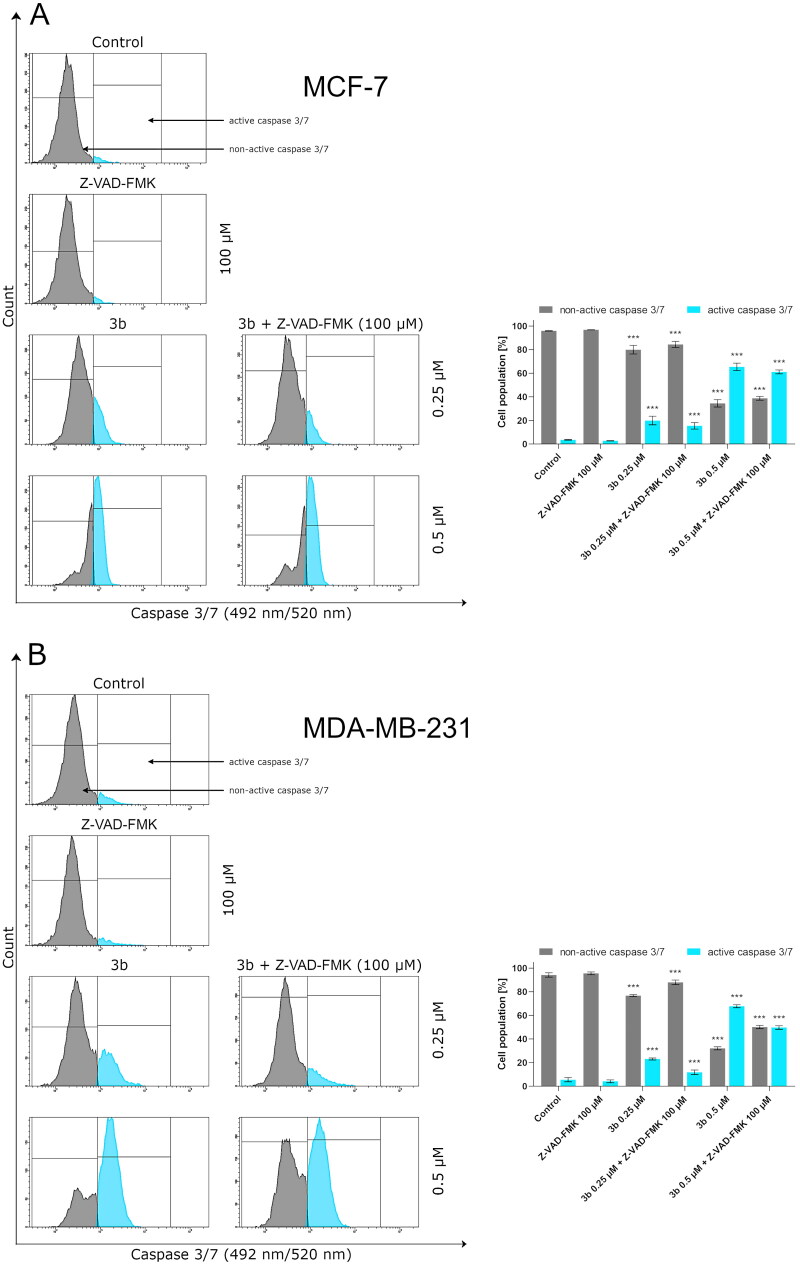
The activity of caspase 3/7 in MCF-7 (A) and MDA-MB-231 (B) breast cancer cells incubated with **3b** (0.25 μM and 0.5 μM) in the absence and presence of Z-VAD-FMK (100 μM) for 24 h. Mean percentage values from three independent experiments done in duplicate are presented. ***p < 0.001 vs. control group.

Protein p53 initiates intrinsic apoptosis pathway involving Bax and caspase 9, 3/7. Caspases play crucial role in apoptosis. They are not only the initiators of the process but also the main executors. The initiator caspases are activated by separate protein complexes. Caspase 10 is activated in death signalling protein (DISC) while caspase 9 in the apoptosome. Both caspases (10 and 9) lead to initiation of executor caspases 3 and 7. Therefore, in this study, we have evaluated the effect of the new derivative (**3b**) on the activation of these initiator and executioner caspases in breast cancer cell lines. The 24 h incubation of MCF-7 and MDA-MB-231 with **3b** induced number of cells with active caspases 9, 10, and 3/7 in both breast cancer cell lines compared to control. There were 12.9 ± 0.8% of cells with active caspase 10 at 0.25 µM concentration and 70.8 ± 1.1% at 0.5 µM in MCF-7. In MDA-MB-231, there was 21.2 ± 1.4% (0.25 µM) and 50.8 ± 3.8% (0.5 µM) cells with active caspase 10 (Figure 8). Moreover, 24 h exposure of cells to compound **3b** was shown to result in an increase in caspase 9 activity at both concentration in oestrogen-independent cell line (MDA-MB-231) and oestrogen dependent (MCF-7) cell line. The highest percentage of active form of caspase 9 was observed at 0.5 µM in MCF-7 and was 72.1 ± 2.3%. While in MDA-MB-231 at the same concentration, 55.9 ± 1.2% of cells had active caspase 9 (Figure 9). The induction of executioner caspases in the MCF-7 is related with activation of caspase 7 since this cell line does not express caspase 3^43^. Despite the lack of expression of caspase 3 in MCF-7, it was observed that 24 h incubation of cells with **3b** leads to increase in activity of executive caspase 7. In MDA-MB-231 cell line, we have also observed increase of cells with active form of caspase 3 and 7. At 0.25 µM concentration, there was 23.8 ± 0.8% cells with active executive caspases and at 0.5 µM there was 68.4 ± 1.2% (Figure 10).

To determine the role of the caspases in the activation of apoptosis by **3b**, we pre-treated MCF-7 and MDA-MB-231 cells with irreversible pan-caspase inhibitor Z-VAD-FMK (50 µM) for 60 min. Following incubation, cells were treated with novel synthesised compound **3b** for 24 h. In all instances, Z-VAD-FMK inhibitor decreased the activation of caspase 10, 9, and 3/7 in response to **3b** in both cancer cell lines (Figures 8–10).

The obtained results revealed that compound **3b** initiation of apoptosis was slightly inhibited by pan-caspase inhibitor Z-VAD-FMK. Similar results were obtained by van der Walt^44^. The researchers showed that *Sutherlandia frutescens* activate apoptosis in malignant melanoma cells which is related with activation of caspases. The use of a caspase inhibitor as in the case of the use of compound **3b** resulted in a decrease in the activity of initiator and execution caspases^44^. The rate of inhibition of caspase activity depended on the type of caspase. These data suggest that compound **3b** may induce apoptosis primarily through a caspase-dependent pathway and partially via caspase independent pathway in MCF-7 and MDA-MB-231 cells.

Many cancers result from failure of the apoptosis induction. The apoptotic signal may be blocked by increase of antiapoptotic molecules such as Bcl-2, IAP, FLIP, or decrease of proapoptotic molecules, i.e. Bax. In this case, autophagy may increase the efficacy of treatment by induction of apoptosis or be even alternative process leading to elimination of tumour cells ^45^. However, it can be also involved in tumour formation. The process of autophagy is responsible for maintaining intracellular homeostasis under stress conditions ^46^. Considering the ambiguous importance of autophagy in cancer treatment, we evaluated the effect of compound **3b** on this process.

**Figure 11.**
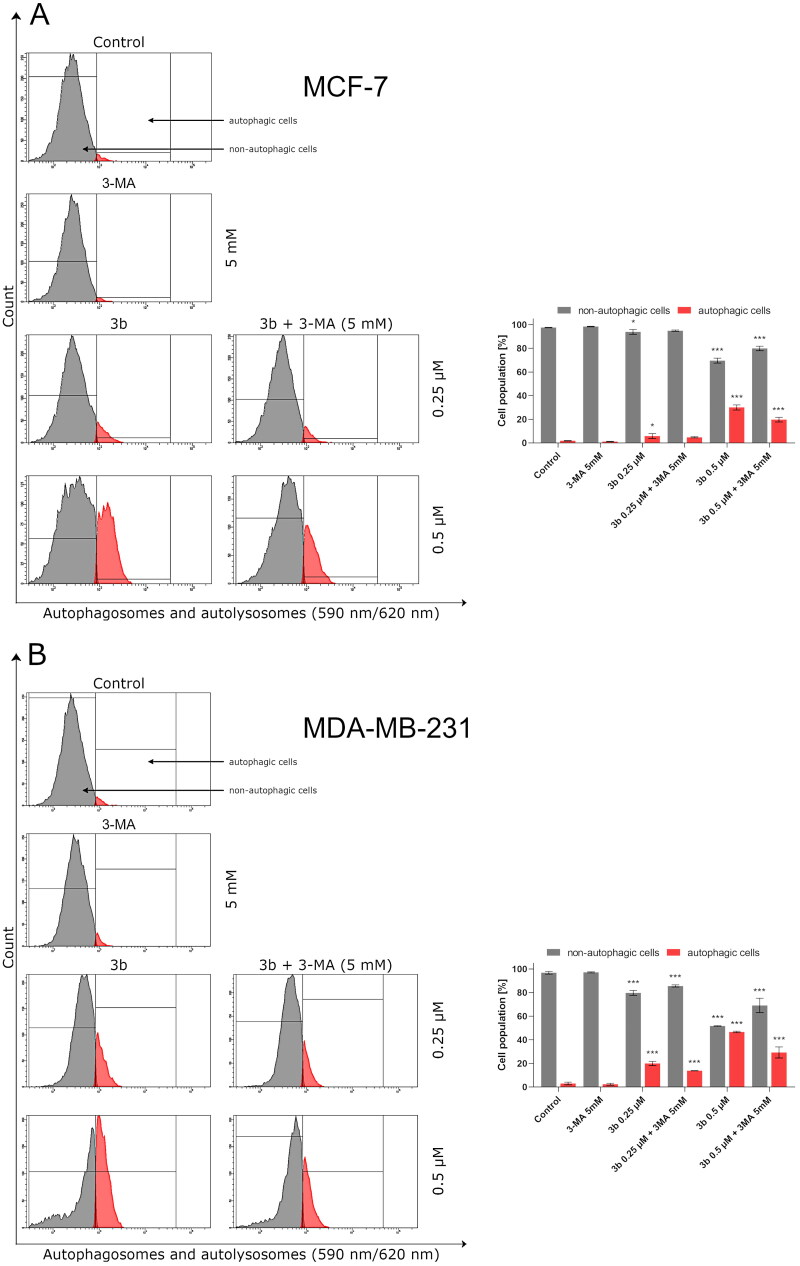
Flow cytometric analysis of autophagy induction in MCF-7 (A) and MDA-MB-231 (B) breast cancer cells incubated with **3b** (0.25 μM and 0.5 μM in the absence or presence of 3-MA (5 mM)). Mean percentage values from three independent experiments done in duplicate are presented. *p < 0.05 vs. control group, ***p < 0.001 vs. control group.

After 24 h incubation, it was observed that **3b** in both concentrations (0.25 µM and 0.5 µM) induced autophagy in MCF-7 and MDA-MB-231 cells. The highest rate of autophagosomes was noted in MDA-MB-231 cells incubated with **3b** at 0.5 µM (47.1 ± 0.6%). In the case of MCF-7 incubated with this compound at same concentration, 30 ± 2.1% autophagic cells were registered. Pre-treatment of MCF-7 and MDA-MB-231 with autophagy inhibitor (3-MA, 1 mM, 1 h) resulted in inhibition of the autophagy in cells treated with **3b**. MDA-MB-231 treated with **3b** (0.5 µM) and 3-MA generated 25.9 ± 4.7% of autophagic cells, while in MCF-7 incubated with **3b** (0.5 µM) + 3MA, there was one and a half times less autophagic cells than in cells incubated with **3b** (0.5 µM). The results are presented in Figure 11.

**Figure 12.**
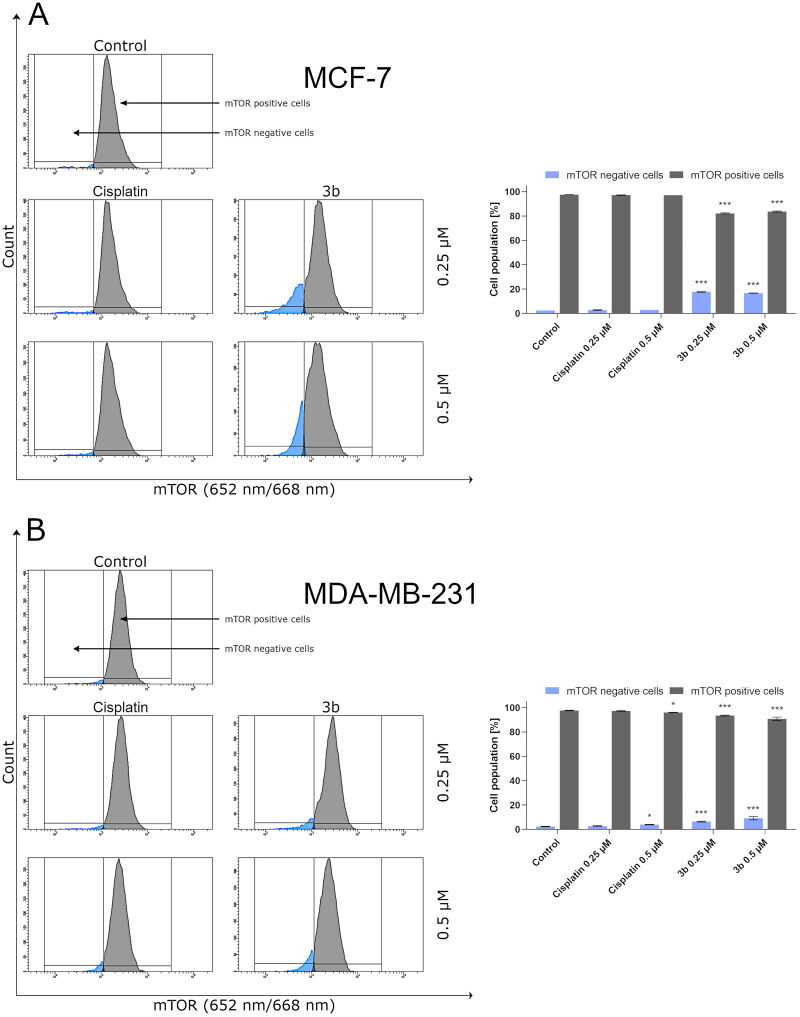
Anti-mTOR antibody flow cytometric analysis of MCF-7 (A) and MDA-MB-231 (B) breast cancer cells compared to a negative control cell after 24 h of incubation with **3b** and cisplatin (0.25 μM and 0.5 μM). Mean percentage values from three independent experiments done in duplicate are presented. *p < 0.05 vs. control group, ***p < 0.001 vs. control group.

It has been reported that overactivation of PI3K/Akt/mTOR signalling pathway is commonly observed in cancer cells ^47^. Based on our previous results, on the induction of production of autophagosomes by **3b** in both breast cancer cell lines, we have evaluated effect of this derivative on mTOR inhibition. It has been shown that compound **3b** at both used concentrations (0.25 µM and 0.5 µM) leads to a decrease in the number of cells with active mTOR (Figure 12).

**Figure 13.**
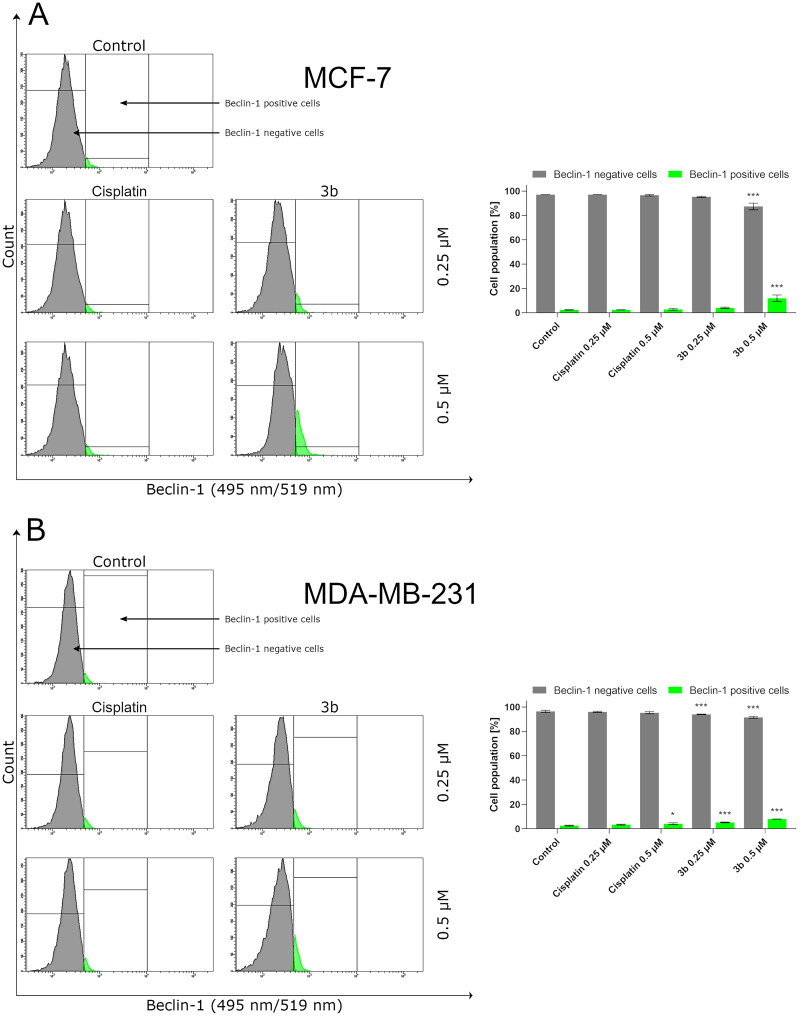
Anti-beclin-1 antibody flow cytometric analysis of MCF-7 (A) and MDA-MB-231 (B) breast cancer cells compared to a negative control cell after 24 h of incubation with **3b** and cisplatin (0.25 μM and 0.5 μM). Mean percentage values from three independent experiments done in duplicate are presented. *p < 0.05 vs. control group, ***p < 0.001 vs. control group.

One of the autophagy marker’s is beclin-1. In our study, we have proved that compound **3b** increases the number of cells with active beclin-1 in dose-dependent manner in both breast cancer cells. In MCF-7 after 24 h incubation with cisplatin and **3b** at 0.5 µM concentration, we observed 4.1 ± 0.5% and 13.9 ± 2.6% cells with active beclin-1, respectively. While in MDA-MB-231, we noted 3.6 ± 0.7% cells with active beclin-1 after treatment with cisplatin (0.5 µM) and 8.1 ± 0.1% in **3b** (0.5 µM). The obtained results are presented in Figure 13.

**Figure 14.**
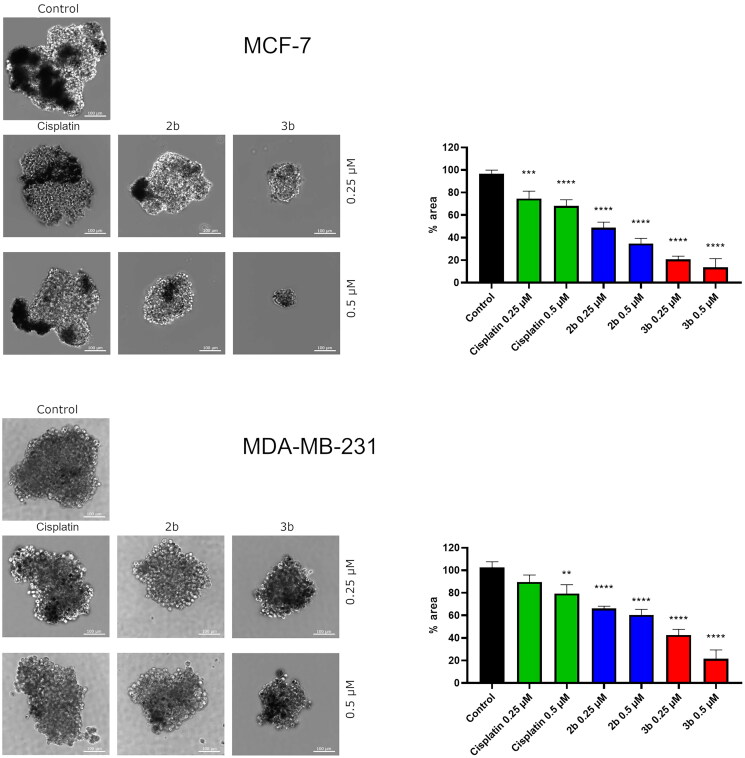
Microscopy images of cell invasion of spheroids incubated for 24 h with **2b**, **3b** and cisplatin (0.25 μM and 0.5 μM). Representative photographs are shown; scale bar: 100 μm. Microscopy images of cell invasion of spheroids incubated for 24 h with **2b**, **3b** and cisplatin (0.25 μM and 0.5 μM). Representative photographs are shown; scale bar: 100 μm. **p < 0.01 vs. control group, ***p < 0.001 vs. control group, ****p < 0.0001 vs. control group.

Recent progress in drug discovery has resulted in 3D *in vitro* culturing tools. Three-dimensional cell culture more closely reflected the conditions in the human body than monolayer 2D. Thus, we investigated the effect of novel compounds **2b** and **3b** on spheroid formation in MCF-7 and MDA-MB-231. The obtained results revealed that both derivatives, the sulphonyl (**2b**) and tetrazole (**3b**), lead to slower formation of spheroids compared to untreated cells. Moreover, the edges of MCF-7 spheroids exposed to **2b** and **3b** were frayed and blurred vs. control. Cisplatin used as reference drug at 0.25 µM and 0.5 µM concentration slightly affected growth of spheroids in MCF-7 and MDA-MB-231. The strongest inhibition of spheroid formation was observed in MCF-7 cells incubated with **3b** at 0.5 µM concentration. The spheroids were approximately 86.37 ± 7.7% smaller compared to the control (Figure 14).

## Discussion

Nowadays, a significant part of scientific research is focused on prevention, early detection, and effective treatment of cancer. According to estimates of GLOBOCON, the number of breast cancer deaths in the world has increased by almost 84% compared to the year 2000^1^^,^^48^. In addition to this, despite the great progress in the treatment of cancer and dynamic development of pharmacology, modern medicine still does not have effective and minimally invasive anticancer therapy. Currently used therapy has low specificity towards cancer cells which causes undesirable side effects. Thus, one of the greatest challenges of the modern oncology is synthesis of effective and safe anticancer drugs for patients. It is worth noting that heterocyclic systems possess variable biological activity and are widely used in pharmaceuticals, antibiotics, and in agrochemicals. In addition to this, almost 75% of FDA-approved drug by contain a nitrogen heterocycle ^49^. Among *N*-heterocycles, 1,2,4-triazine derivatives present an interesting group of anticancer drugs. Currently, numerous studies are underway to utilise this moiety in designing the most optimal lead structure with anticancer activity^11^^,^^13–15^^,^^50–52^. In 2020, one of the derivatives of 1,2,4-triazine was approved in cancer treatment. Blueprint Medicines Corporation registrated Ayvakit in gastrointestinal stromal tumours (GIST). The active substance of this drug is avapritinib which is a kinase inhibitor of PDGFRα ^53^. The high efficacy of the compound in comparison to other tyrosine kinase inhibitors has led to further clinical trial using this compound in the treatment of other advanced solid tumours such as lung, breast, melanoma, and sarcoma^54^^,^^55^.

Nevertheless, important strategy in the search for chemotherapeutics is the approach based on combining fragments of known drugs in a single molecule. It is intended to design compounds with increased biological activity than the original compounds. Combining natural pyrazolo[4,3-*e*][1,2,4]triazine system with pharmacophore groups provides an opportunity to design new chemical compounds with potential anticancer activity. Mojzych et al. presented synthesis of aza-sildenafil analogues and aniline substituted pyrazolo[4,3-*e*][1,2,4]triazine sulphonamides. These compounds exerted moderate antitumor activity in breast cancer cells and potential mechanism of action was associated with inhibition of tumour associated carbonic anhydrase isoforms (IX and XII) ^56^. In 2010, Gucký et al. presented series of pyrazolo[4,3-*e*][1,2,4]triazine derivatives which exerted cytotoxic activity against five cancer cell lines (T-lymphoblastic leukaemia, T-lymphoblastic leukaemia daunorubicin-resistant clone, myeloid leukaemia, myeloid leukaemia paclitaxel resistant clone, and lung adenocarcinoma)^57^. As can be seen, the incorporation of a sulphonamide group to pyrazolotriazine moiety leads to an increase in the antitumor activity of the original compounds. In addition to this, the structure of pyrazolo[4,3-*e*][1,2,4]triazine can be modified by incorporation of tetrazole or triazole ring. A 2011 study showed that the insertion of a terminal tetrazole or triazole ring in replacement of SCH_3_ group resulted in a significant increase in cytotoxic activity against breast cancer cell line. For the triazole derivative, the IC_50_ value is 4 µM while for tetrazole it is 0.5 µM ^58^. These premises prompted our research team to use pyrazolo[4,3-*e*]tetrazolo[1,5-*b*][1,2,4]triazine system conjugated with sulphonamide group to synthesise new derivatives with potential biological activity^12–14^^,^^51^. Our previous studies on bioactive compounds of pyrazolo[4,3-*e*]tetrazolo[1,5-*b*][1,2,4]triazine derivatives ^13^^,^^14^^,^^51^ against colon cancer cell lines were very promising. In view of high anticancer activity of these compounds and exploiting literature on the synthesis and functionalisation of the hitherto little known pyrazolo[4,3-*e*][1,2,4]triazine system, we designed novel compounds **2a**, **2b**, **3a**, and **3b**. Chlorosulfonyl derivative (**1**) in reaction with amines led to sulphonyl derivatives (**2a,b**), which were used in a nucleophilic substitution reaction of methylsulfonyl group with sodium azide. The reaction directly led to the final tetrazolium derivatives (**2a**,**b**). Based on conducted experiments, the new synthetic derivatives **2a**, **2b**, **3a**, and **3b** have shown high cytotoxic activity against MCF-7 and MDA-MB-231 breast cancer cells in the micromolar concentration range (IC_50_ from 0.25 µM to 9.2 µM). Moreover, synthesised compounds indicated low cytotoxic effect on normal cell lines. The nucleophilic substitution of SO_2_CH_3_ group with sodium azide was shown to yield tetrazolium derivatives of pyrazolo[4,3-*e*][1,2,4]triazine (**3a,b**) with stronger cytotoxic properties than sulphonamide derivatives (**2a,b**). Compound **3a** was 17 times stronger than **2a** against MCF-7 and almost 13 times against MDA-MB-231. Compound **3b** was 22 times stronger than **2b** in MCF-7 and 25 times against MDA-MB-231. It is worth mentioning that all synthesised compounds have notable higher cytotoxic activity than reference drug (cisplatin). Czarnomysy et al. reported that IC_50_ of cisplatin of MCF-7 cells was 93 µM and 82 µM of MDA-MB-231 cells after 24 h incubation^31^. Preliminary MTT assay results allowed us to select **2b** as most active sulphonyl derivative and **3b** as the most active tetrazole derivative.

Research on the effect of drug chirality on their pharmacological activity has been conducted for many years. The differences in the pharmacological activity of particular isomers may have serious consequences for the treatment. For this reason, stereochemistry has become an important part of the drug development. Chiral biologically active compounds can be classified into one of the following categories: both enantiomers exhibit favourable pharmacological activity, only one of the enantiomers is pharmacologically active, both enantiomers have desirable pharmacological activity but different mechanism and one of the enantiomers has therapeutic effect and the other acts as an antagonist^59^^,^^60^. In our studies, we have proved that both pairs of enantiomers **2ab** and **3ab** have similar cytotoxic activity against breast cancer cell lines (MCF-7 and MDA-MB-231) and normal cells (MCF-10A). However, the *R*-enantiomers of synthesised compounds exerted slightly higher anticancer activity than their respective *S*-isomers.

Currently, approaches utilised in cancer chemotherapy are likely to affect the apoptosis^61^. Thus, to compare biological activity of sulphonamide derivative (**2b**) and its corresponding tetrazolium derivative (**3b**), their effect on the molecular mechanism of apoptosis in both breast cancer cells was evaluated. The study revealed that both **2b** and **3b** derivatives have proapoptotic effect against MCF-7 and MDA-MB-231. Recent research indicates that exposure of cancer cells to chemotherapeutics induces apoptosis mediated by ROS^27^. Increased level of ROS by drugs may be due to mitochondrial generation of ROS and depletion of antioxidant systems. ROS is engaged in activation of p53, JNK, and proapoptotic Bcl-2 proteins (i.e. Bax) and oxidation of cardiolipin which induce release of cytochrome C to cytosol and initiate intrinsic pathway of apoptosis. Moreover, ROS may also trigger apoptosis through extrinsic pathway by transmembrane death receptors (i.e. TNFR, TRAIL)^62^^,^^63^. The links between ROS and apoptosis prompted us to evaluate the effect of **3b** on the level of ROS after 24 h incubation in MCF-7 and MDA-MB-231 breast cancer cells. Our results confirmed the previous research reports^27^^,^^64^^,^^65^, that anticancer activity of chemotherapeutics may be related with induction of reactive oxygen species. Compound **3b** at 0.5 µM increased ROS level in MCF-7 12 times and four times in MDA-MB-231 compared to respective control.

As it was mentioned, programmed cell death is related with morphological and biochemical modifications which embrace upregulation of Bax, p53, and caspases^66^. To identify that apoptosis induced by **3b** flow cytometry methods were used (activity levels of caspase 9, 10, 3/7 and protein activation p53, Bax), the obtained results revealed that novel synthesised compound **3b** induces the level of Bax and p53 proteins in both breast cancer cell lines in dose-dependent manner. Our findings are consistent with those of Zaki et al. who synthesised 1,2,4-triazinone derivatives and proved that these compounds induce apoptosis in MCF-7 breast cancer cell by increasing level of proteins involved in intrinsic pathway of programmed cell death, i.e. Bax and p53^67^.

One of the main enzymes engaged in apoptosis are caspases. They participate by their cysteine moiety in the catalytic process and the digestion of the proteins directly after asparagine^68^. Keeping this in view, we have proved that compound **3b** induces apoptosis by activation of cascade caspase. This results present promising strategy for the treatment of cancer. However, to understand the role of caspases in the induction of apoptosis by **3b** in MCF-7 and MDA-MB-231, pan-caspase inhibitor Z-VAD-FMK was used. We confirmed that Z-VAD-FMK inhibitor slightly reduced the number of cells with active caspase −3/7, −9, and −10 indicating that caspase may be a potential molecular mechanism of **3b**. The decrease in caspase activity was more significant in MDA-MB-231 cells than in MCF-7 cells.

One of the significant links between cancer progression and cell death is NF-κB. It is nucleus of B cells that is the transcriptional factor. In the cancer cells, overexpression of NF-κB is observed which suppresses programmed cell death by exciting expression of IAP, cFLIP, and TNF – inhibitory genes^69^. Recent scientific reports of *in vitro* studies suggest inhibition of NF-κB may sensitise cancer cells to chemotherapy which occur by launching apoptosis in these cells. Bortezomib in head and neck squamous cell carcinoma^70^ has anticancer effect by inhibition of NF-κB, activation of apoptosis and inhibition of proliferation of cancer cells. The results presented in this paper indicate that compound **3b** also inhibits the number of cells with active NF-κB.

According to the recommendations of the Nomenclature Committee on Cell Death (NCDD) besides apoptosis, other types of cell death have been distinguished including autophagy^71^. Nowadays, the role of autophagy in development of cancer is not clear^72^. To evaluate whether the mechanism of action of novel pyrazolo[4,3-*e*]tetrazolo[1,5-*b*][1,2,4]triazine on the breast cancer cells is related to autophagic response, we measured the number of autophagosomes and level of beclin-1 after 24 h incubation with compound **3b**. We found that **3b** stimulates autophagy process in MCF-7 and MDA-MB-231 by inducing autophagosome formation and induction of beclin-1 level in dose-dependent manner. In addition to this, we investigated the possibility that 3-MA could inhibit autophagy induced by **3b**. Co-treatment with autophagy inhibitor (3-MA) resulted in decrease of formation of autophagosomes in both cancer cell lines. As the concentration of **3b** increased, the proautophagic effect was more noticeable. The role of autophagy in cancer is still inconclusive. However, many scientific reports indicate that autophagy is a basis for anticancer activity^25^^,^^27^^,^^72^^,^^73^. One of the factors involved in either apoptosis or autophagy is mTOR. Overexpression of PI3K-Akt-mTOR pathway induced the oncogenesis in various cancers: breast, gastric, melanoma, and hepatocellular ^74^. Increased activity of this pathway is a common result of mutations observed in cancer cells. Hence, inhibition of this signalling pathway not only induces apoptosis in cancer cells but also stimulates autophagy. We discovered that incubation with **3b** downregulates the number of cells with active mTOR in a dose dependent manner in both breast cancer cell lines at 24 h. These results indicate that the pyrazolo[4,3-*e*][1,5-*b*][1,2,4]triazine derivative (**3b**) might induce autophagy via PI3K/AKT/mTOR pathway.

Another protein required for the initiation of autophagy is beclin 1. Under physiological conditions, this protein remains in complex with the anti-apoptotic proteins Bcl-2 and Bcl-xl. Under the influence of stress factors, e.g. chemotherapeutics beclin 1 is released from the complex and induce formation of autophagosomes^75^^,^^76^. Our research showed that compound **3b** slightly induced the level of beclin 1 in both breast cancer cell lines.

The intermediate form between monolayer and *in vivo* is multicellular tumour spheroids. Morphologically co-cultures in spheroids are composed of the cells with different phenotypes, proliferating cells, and necrotic cells located in the middle of spheroid. The responses of spheroids and tumours growing *in vivo* to administrated antitumor agents are similar^77–81^. According to this, we proved that novel synthesised compounds **2b** and **3b** reduced the size of MCF-7 and MDA-MB-231 spheroids. The strongest inhibition of spheroid formation in both breast cancer cell lines was caused by compound **3b** at 0.5 µM.

## Conclusions

We have proved that new sulphones (**2ab**) and tetrazole derivatives (**3ab**) have potent anticancer activity against breast cancer. It was shown that new pairs of enantiomer derivatives **2a,b** and **3a,b** possess cytotoxic properties against MCF-7 and MDA-MB-231 in the micromolar concentration range (IC_50_ 0.25 µM from to 6.0 µM). In addition, novel synthesised compounds have less effect on the survival of normal cells: MCF-10A. Preliminary MTT assay results allowed us to select *R*-enantiomers for further more detailed biological research. Studies on molecular mechanism of action of **2b** and **3b** revealed that both synthesised compounds lead to initiation of apoptosis in two breast cancer cell lines. This enabled compound **3b** to be selected as the most active. Compound **3b** induced apoptosis through activation of extrinsic and intrinsic pathway. This compound increased the activity of p53, Bax, and ROS and decreased NF-κB which play crucial role in activation of apoptosis. Moreover, the compound **3b** decreased the level of mTOR which play main role in autophagy. It is known that process of autophagy is impaired in cancer cells. The results revealed that **3b** induces formation of autophagosomes in MCF-7 and MDA-MB-231. Preincubation of cells with an autophagy inhibitor (3-MA) caused inhibition of **3b** induced autophagosome formation. Beclin-1 also plays a crucial role in the process of autophagy. We proved our novel compound **3b** increased the level of this protein in both breast cancer cells, but the effect was negligible. It suggests that activation of autophagy by **3b** via beclin-1 is minor. The inhibitory effect of 3-MA was more prominent in cells incubated with the compound **3b** at a concentration of 0.5 µM. The obtained results extend the existing knowledge on the molecular mechanism of anticancer activity of pyrazolo[4,3-*e*]tetrazolo[1,5-*b*][1,2,4-triazine] derivatives. Further studies to understand the molecular pathways involved in the anticancer activity and effect of the described group of compounds on the expression of genes involved in the apoptosis and autophagy are necessary and will be continued.

## Supplementary Material

Supplemental Material

## Data Availability

Department of Synthesis and Technology of Drugs, Medical University of Bialystok, Kilinskiego 1, 15-089 Bialystok, Poland.
