# Supplementary material for: The activity of pyrazolo[4,3-e][1,2,4]triazine and pyrazolo[4,3-e]tetrazolo[1,5-b][1,2,4]triazine sulphonamide derivatives in monolayer and spheroid breast cancer cell cultures
Source: J Enzyme Inhib Med Chem. 2024 May 3;39(1):2343352. doi: 10.1080/14756366.2024.2343352 (PMC11073428; doi:10.1080/14756366.2024.2343352)
Supplement: Supplemental Material [file IENZ_A_2343352_SM0236.pdf]

Compound 2a

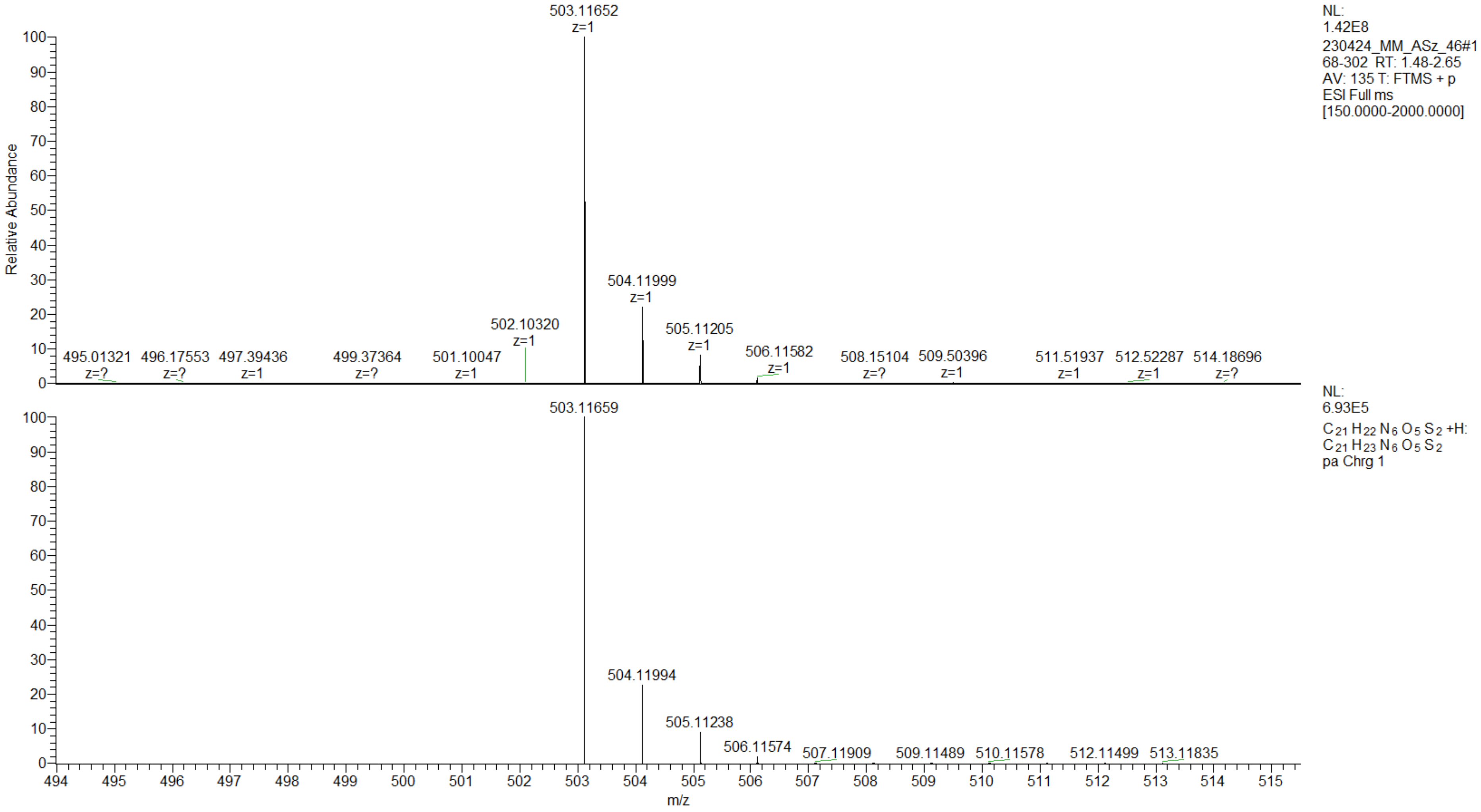

503.11652
z=1
504.11999
z=1
502.10320
z=1
505.11205
z=1
506.11582
z=1
495.01321
z=?
496.17553
z=?
497.39436
z=1
499.37364
z=?
501.10047
z=1
508.15104
z=?
509.50396
z=1
511.51937
z=1
512.52287
z=1
514.18696
z=?
NL:
1.42E8
230424_MM_ASz_46#1
68-302 RT: 1.48-2.65
AV: 135 T: FTMS + p
ESI Full ms
[150.0000-2000.0000]
Relative Abundance
503.11659
504.11994
505.11238
506.11574
507.11909
509.11489
510.11578
512.11499
513.11835
NL:
6.93E5
C 21 H 22 N 6 O 5 S 2 +H:
C 21 H 23 N 6 O 5 S 2
pa Chrg 1
m/z

Compound 2b

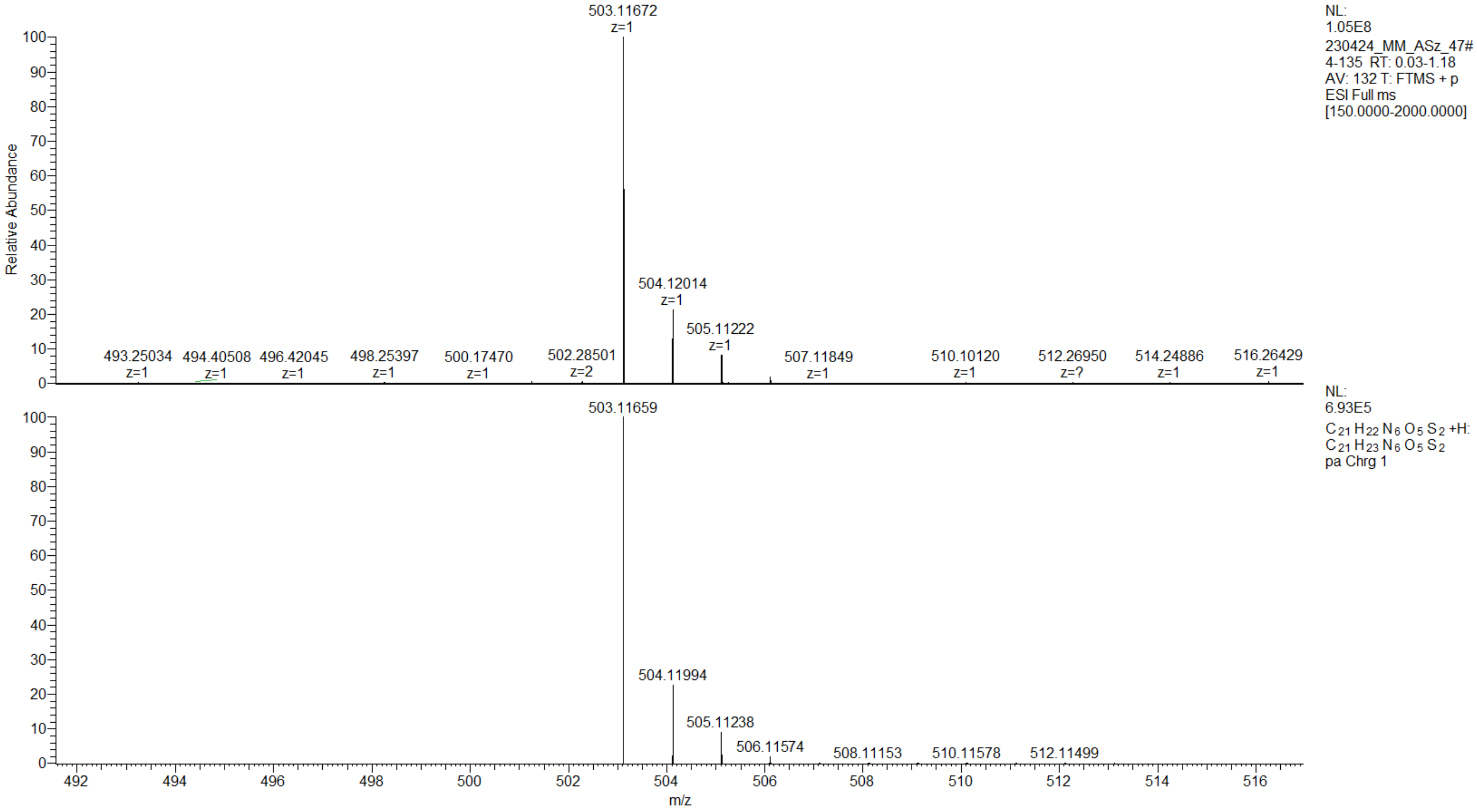

503.11672
z=1
NL:
1.05E8
230424_MM_ASz_47#
4-135 RT: 0.03-1.18
AV: 132 T: FTMS + p
ESI Full ms
[150.0000-2000.0000]
Relative Abundance
504.12014
z=1
505.11222
z=1
493.25034
z=1
494.40508
z=1
496.42045
z=1
498.25397
z=1
500.17470
z=1
502.28501
z=2
507.11849
z=1
510.10120
z=1
512.26950
z=?
514.24886
z=1
516.26429
z=1
NL:
6.93E5
503.11659
C 21 H 22 N 6 O 5 S 2 +H:
C 21 H 23 N 6 O 5 S 2
pa Chrg 1
504.11994
505.11238
506.11574
508.11153
510.11578
512.11499
m/z

Compound 3a

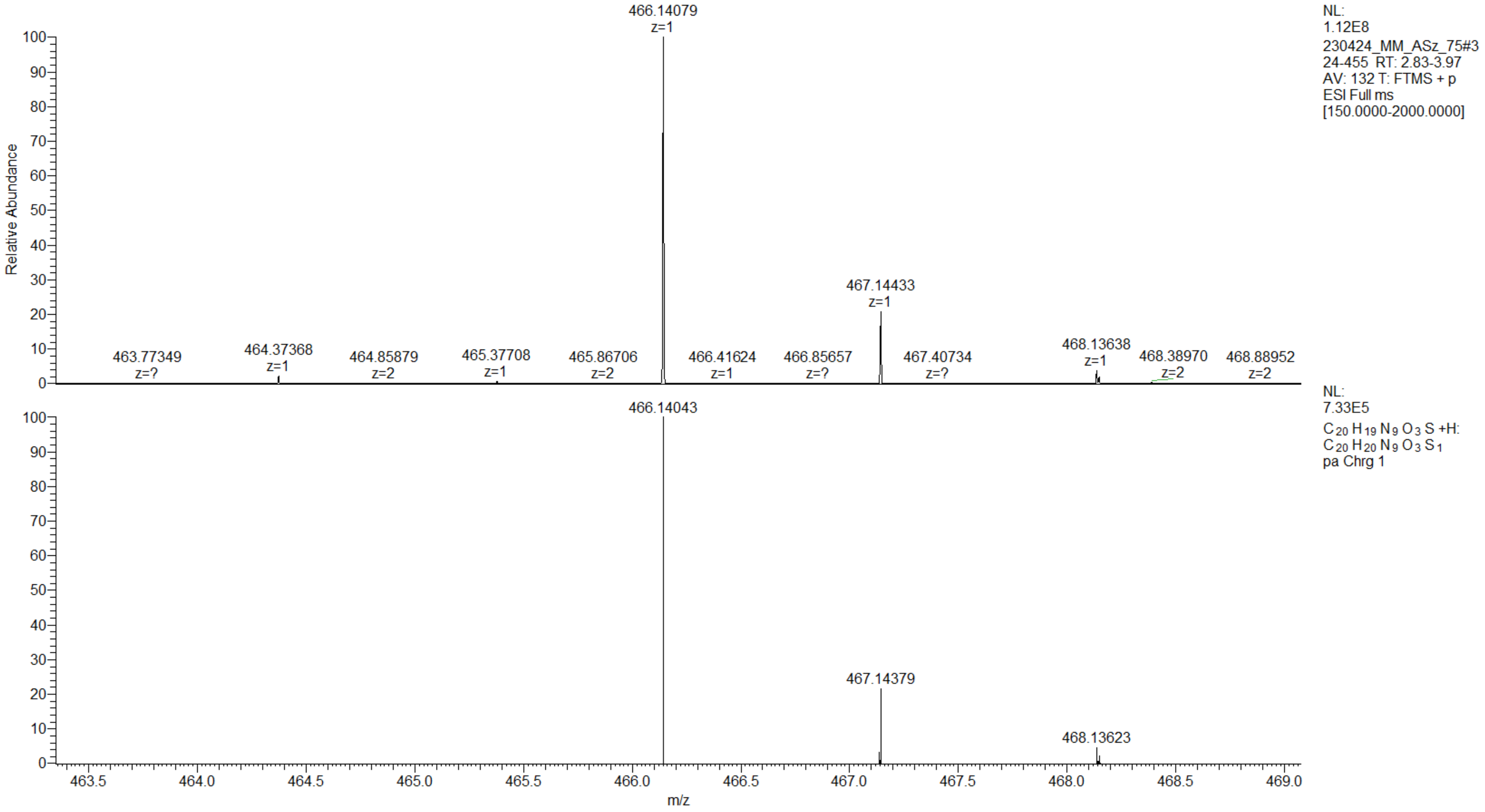

466.14079
z=1
NL:
1.12E8
230424_MM_ASz_75#3
24-455 RT: 2.83-3.97
AV: 132 T: FTMS + p
ESI Full ms
[150.0000-2000.0000]
Relative Abundance
463.77349
z=?
464.37368
z=1
464.85879
z=2
465.37708
z=1
465.86706
z=2
466.41624
z=1
466.85657
z=?
467.14433
z=1
467.40734
z=?
468.13638
z=1
468.38970
z=2
468.88952
z=2
NL:
7.33E5
C 20 H 19 N 9 O 3 S +H:
C 20 H 20 N 9 O 3 S 1
pa Chrg 1
466.14043
467.14379
468.13623
m/z

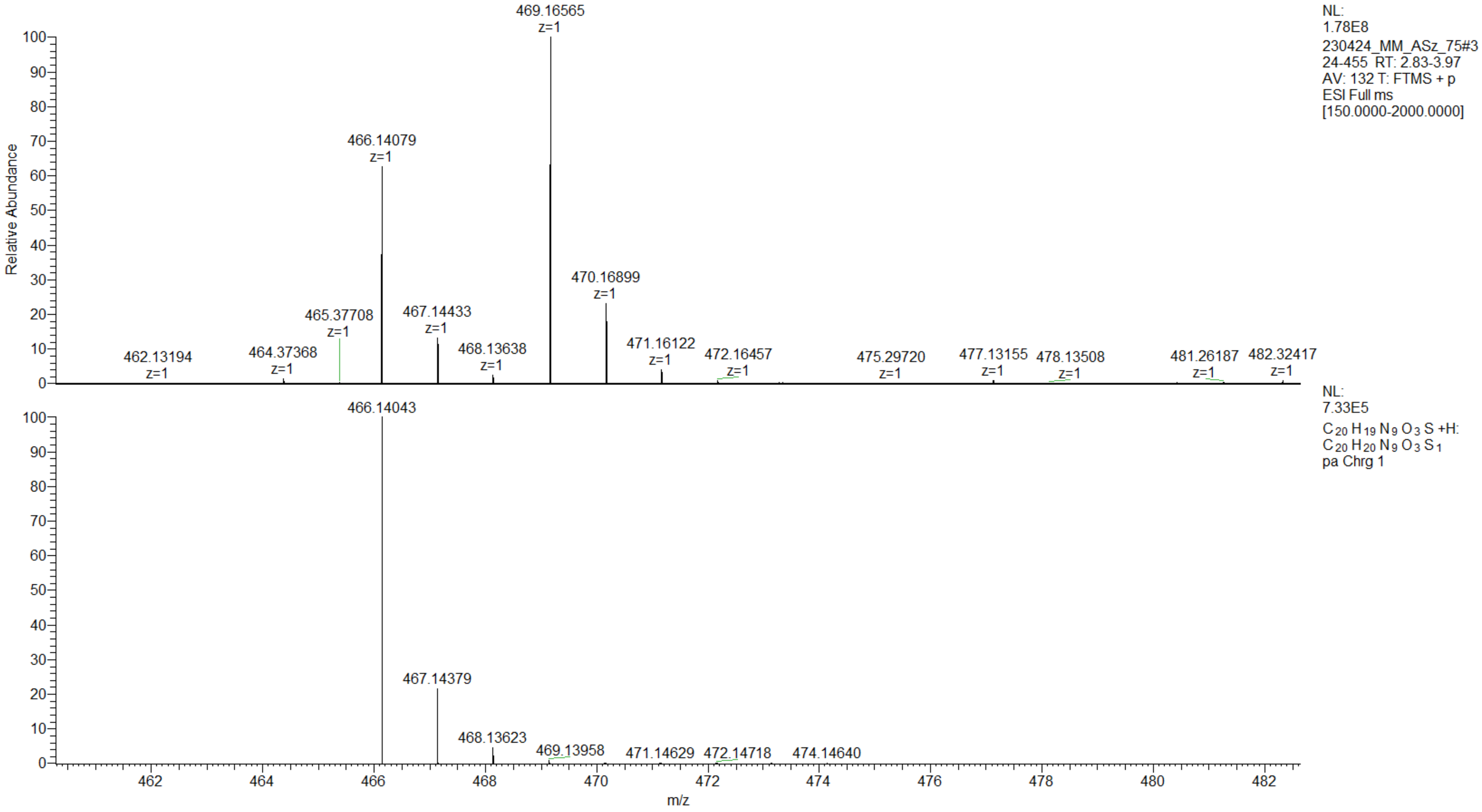

469.16565
z=1
466.14079
z=1
470.16899
z=1
465.37708
z=1
467.14433
z=1
468.13638
z=1
471.16122
z=1
462.13194
z=1
464.37368
z=1
472.16457
z=1
475.29720
z=1
477.13155
z=1
478.13508
z=1
481.26187
z=1
482.32417
z=1
Relative Abundance
NL:
1.78E8
230424_MM_ASz_75#3
24-455 RT: 2.83-3.97
AV: 132 T: FTMS + p
ESI Full ms
[150.0000-2000.0000]
466.14043
467.14379
468.13623
469.13958
471.14629
472.14718
474.14640
NL:
7.33E5
C 20 H 19 N 9 O 3 S +H:
C 20 H 20 N 9 O 3 S 1
pa Chrg 1
m/z

Compound 3b

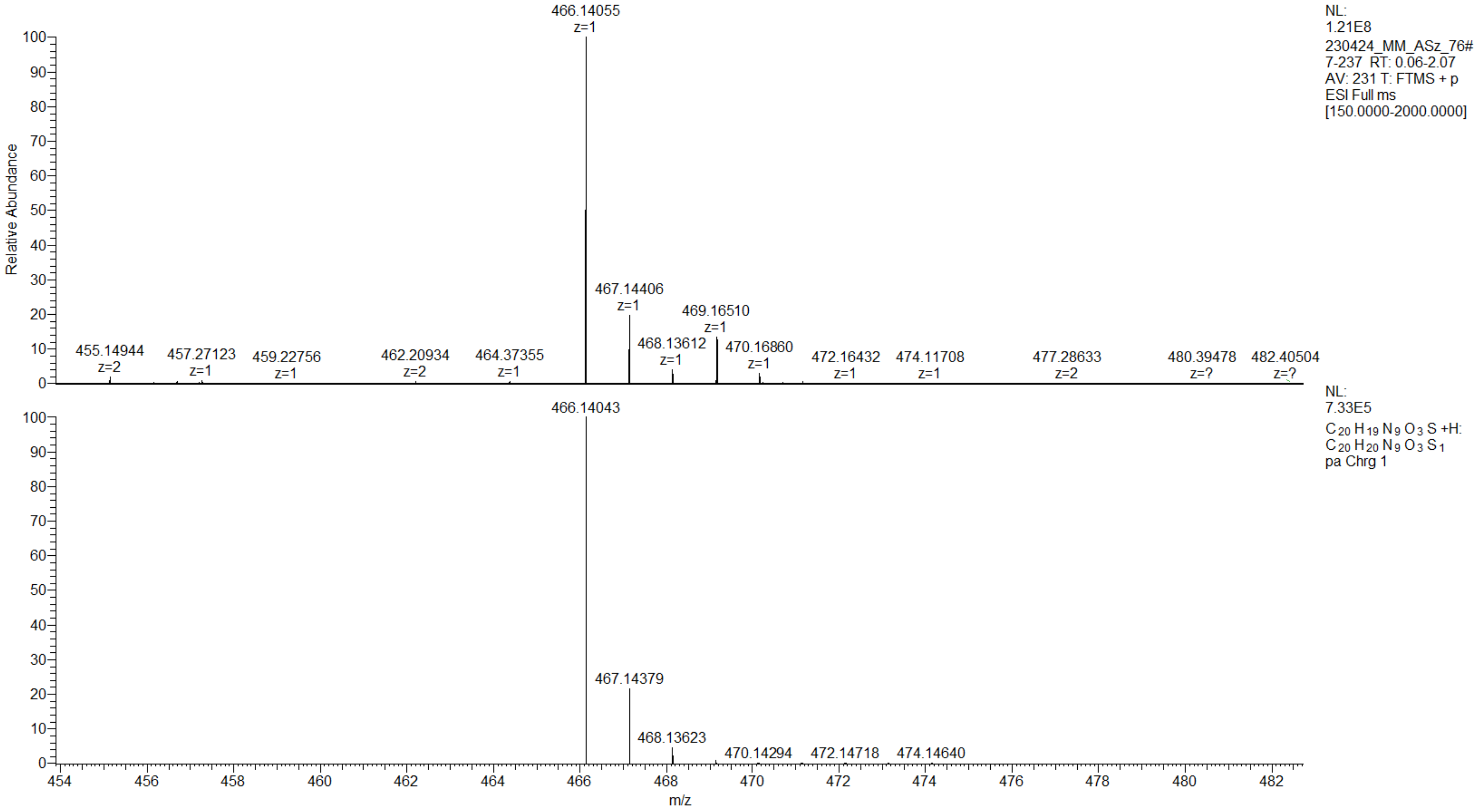
466.14055
z=1
467.14406
z=1
469.16510
z=1
468.13612
z=1
470.16860
z=1
455.14944
z=2
457.27123
z=1
459.22756
z=1
462.20934
z=2
464.37355
z=1
472.16432
z=1
474.11708
z=1
477.28633
z=2
480.39478
z=?
482.40504
z=?
NL:
1.21E8
230424_MM_ASz_76#
7-237 RT: 0.06-2.07
AV: 231 T: FTMS + p
ESI Full ms
[150.0000-2000.0000]
466.14043
467.14379
468.13623
470.14294
472.14718
474.14640
NL:
7.33E5
C 20 H 19 N 9 O 3 S +H:
C 20 H 20 N 9 O 3 S 1
pa Chrg 1
Relative Abundance
m/z
454
456
458
460
462
464
466
468
470
472
474
476
478
480
482
